# Cancer-associated fibroblast-secreted FGF7 as an ovarian cancer progression promoter

**DOI:** 10.1186/s12967-024-05085-y

**Published:** 2024-03-15

**Authors:** Songwei Feng, Bo Ding, Zhu Dai, Han Yin, Yue Ding, Sicong Liu, Ke Zhang, Hao Lin, Zhongdang Xiao, Yang Shen

**Affiliations:** 1 Department of Obstetrics and Gynaecology, Zhongda Hospital, School of Medicine, Southeast University, Nanjing, China; 2 State Key Laboratory of Bioelectronics, School of Biological Science and Medical Engineering, Southeast University, Nanjing, China; 3 Department of Clinical Science and Research, Zhongda Hospital, School of Medicine, Southeast University, Nanjing, China

**Keywords:** Ovarian cancer, FGF7, Epithelial-mesenchymal transition, CAFs, FGFR2

## Abstract

**Background:**

Ovarian cancer (OC) is distinguished by its aggressive nature and the limited efficacy of current treatment strategies. Recent studies have emphasized the significant role of cancer-associated fibroblasts (CAFs) in OC development and progression.

**Methods:**

Employing sophisticated machine learning techniques on bulk transcriptomic datasets, we identified fibroblast growth factor 7 (FGF7), derived from CAFs, as a potential oncogenic factor. We investigated the relationship between FGF7 expression and various clinical parameters. A series of in vitro experiments were undertaken to evaluate the effect of CAFs-derived FGF7 on OC cell activities, such as proliferation, migration, and invasion. Single-cell transcriptomic analysis was also conducted to elucidate the interaction between FGF7 and its receptor. Detailed mechanistic investigations sought to clarify the pathways through which FGF7 fosters OC progression.

**Results:**

Our findings indicate that higher FGF7 levels correlate with advanced tumor stages, increased vascular invasion, and poorer prognosis. CAFs-derived FGF7 significantly enhanced OC cell proliferation, migration, and invasion. Single-cell analysis and in vitro studies revealed that CAFs-derived FGF7 inhibits the ubiquitination and degradation of hypoxia-inducible factor 1 alpha (HIF-1α) via FGFR2 interaction. Activation of the FGF7/HIF-1α pathway resulted in the upregulation of mesenchymal markers and downregulation of epithelial markers. Importantly, in vivo treatment with neutralizing antibodies targeting CAFs-derived FGF7 substantially reduced tumor growth.

**Conclusion:**

Neutralizing FGF7 in the medium or inhibiting HIF-1α signaling reversed the effects of FGF7-mediated EMT, emphasizing the dependence of FGF7-mediated EMT on HIF-1α activation. These findings suggest that targeting the FGF7/HIF-1α/EMT axis may offer new therapeutic opportunities to intervene in OC progression.

**Supplementary Information:**

The online version contains supplementary material available at 10.1186/s12967-024-05085-y.

## Introduction

Ovarian cancer (OC) is a formidable malignancy characterized by its aggressive nature and limited treatment options [1]. Recent research has revealed the significant influence of the tumor microenvironment (TME) on OC progression, particularly the role of cancer-associated fibroblasts (CAFs) in driving tumor growth, invasion, and metastasis [2]. Understanding the molecular mechanisms underlying CAFs-mediated effects on OC cells is crucial for developing effective therapeutic strategies.

Bioinformatics analysis of large-scale datasets has proven invaluable in identifying key genes involved in the crosstalk between CAFs and cancer cells [3–6]. Utilizing this approach, several studies have uncovered potential oncogenic factors originating from CAFs. For instance, Galbo et al. [7] conducted a comprehensive analysis across various cancer types, identifying six distinct subtypes of CAFs characterized by unique molecular features and genetic pathways. These subtypes exhibited varying clinical outcomes and showed involvement in immunotherapy resistance, suggesting the potential for subtype-targeted therapies and underscoring their significance in cancer pathogenesis and prognosis. In a study by Hegab et al. [8], the role of CAFs and the FGFs/FGFR signaling pathway in lung adenocarcinoma was investigated. Their findings demonstrated that CAFs facilitate tumor growth by secreting FGF2 and promoting the conversion of tumor-associated macrophages (TAMs) into the M2 phenotype, which supports tumor progression. Additionally, Eckert et al. [9] employed a label-free proteomic workflow to explore the proteogenomics of high-grade serous carcinoma, focusing on both the tumor and stromal compartments. They discovered that the methyltransferase nicotinamide N-methyltransferase (NNMT) played a crucial role in the CAF phenotype, influencing cytokine secretion, and extracellular matrix production.

Additionally, the bioinformatics analysis and experiments of CAFs-derived genes has identified other key players involved in the crosstalk between CAFs and OC cells. These genes include cytokines, chemokines, extracellular matrix components, and receptors [2]. Studies have highlighted the significance of transforming growth factor beta (TGF-β) signaling in promoting OC progression through its interaction with CAFs [10, 11]. Activation of the c-Met/PI3K/Akt and GRP78 signaling pathways by CAFs-derived hepatocyte growth factor (HGF) promotes cell proliferation in OC cell lines [12]. The secretion of epidermal growth factor (EGF) by CAFs maintains ITGA5 expression and sustains aggregates formed by CAFs and ascites tumor cells [13]. Moreover, EGF from CAFs can promote the early peritoneal dissemination of high-grade serous ovarian cancer (HGSOC), thereby accelerating the development of ascites [13]. Despite the increasing body of research elucidating the role of CAFs in tumor progression through the secretion of various factors, the complete understanding of the intricate molecular regulatory mechanisms governing CAFs is still lacking.

Here, our study presents a comprehensive investigation into the key genes (FGF7) identified through bioinformatics analysis of the transcriptomic landscape of CAFs and OC tissues. To further investigate the molecular mechanisms underlying CAFs-mediated effects on OC cells, functional experiments combining single-cell transcriptome analysis and in vitro assays have been employed. These studies have provided insights into the intricate signaling pathways and molecular interactions between CAFs-derived genes and OC cells.

## Methods

### Isolation of CAFs and NFs

Tumor tissue specimens were pathologically confirmed as HGSOC, while the normal ovarian epithelium tissues from patients undergoing total hysterectomy with bilateral salpingectomy due to benign disease (uterine fibroids) was confirmed as having no invasion by two pathologists from the Zhongda Hospital, Southeast University. Briefly, to extract CAFs and normal fibroblasts (NFs), we collected tissues from March 2022 to November 2022, and used collagenase IV (Invitrogen, CA, USA) to dissociate cells for 2–3 h at 37 °C [14]. These tissues are subjected to enzymatic digestion, and the resulting cells are similarly purified using 70 µm mesh (BD Falcon, USA). The filtrate was subjected to centrifugation, followed by cell culture in red blood cell lysis buffer to remove erythrocytes, and subsequent washing of the resulting cells with phosphate-buffered saline (PBS). When the isolated cultured cells reached a fusion rate of over 80%, an appropriate amount of trypsin was added to observe changes in cell morphology. Fibroblasts are more sensitive to trypsin, thus they are digested first before tumor cells. Subsequently, half of the digestion solution was immediately transferred to another culture flask and supplemented with an appropriate amount of complete culture medium to continue culturing. This gradient digestion method was employed for every subculture, typically for 3–4 passages, until the purity of fibroblasts reached above 95% for subsequent experiments.

To prepare conditioned media (CM) from fibroblast cultures, fibroblasts were allowed to reach 70 to 80% confluency in the culture flask. Subsequently, the medium containing fetal bovine serum was replaced with a serum-free medium. The cultures were then incubated for an additional 48 h, following which the supernatant was collected. This supernatant was centrifuged at 3000 rpm for 10 min at room temperature to remove cellular debris. The cell count from the remaining culture was determined to standardize the conditioned medium. The serum-free medium was adjusted to a final concentration of 1 ml of conditioned medium per 1 × 10^6^ cells. For the preparation of cancer-associated fibroblasts-conditioned medium (CAFs-CM) and normal fibroblasts-conditioned medium (NFs-CM), 10% fetal bovine serum (FBS) was added to the standardized medium. The resulting conditioned medium can be stored at either 4 °C or − 80 °C for subsequent experiments.

### Cell lines and cell culture

The A2780, Human Umbilical Vein Endothelial Cells (HUVEC), and HO8910 cell lines were obtained from Dr. Cheng at the School of Medicine and Holistic Integrative Medicine, Nanjing University of Chinese Medicine. Authentication of all cell lines was performed through short tandem repeat (STR) typing. Both the human cell line and fibroblasts were cultured in DMEM/F12 medium (KGM12500S-500, keyGEN, China) supplemented with 10% fetal bovine serum (FBS) and 1% penicillin/streptomycin. Culturing conditions involved maintaining the cells at 37 °C with 5% CO_2_ and 95% air. To stimulate in vitro, hFGF7 (10210-H07E, Sino Biological, China) was added to the medium at concentrations of 10 ng/ml and 20 ng/ml for 48 h of incubation. For inhibitor studies, a 5 μM concentration of FGFR2 antagonist (AZD4547), which is highly sensitive to FGFR2, was used according to the specifications [15]. In neutralization experiments, the CM was treated with 1 μg/ml of FGF7 neutralizing antibody (MAB251-SP, R&D Systems, USA) for 4 h prior to use. Concentrations of 10 μM concentration of MG-132 was added to medium for 3, 6 or 9 h.

### Western blotting

Western blotting was conducted in accordance with previously described standard protocols [16]. Primary antibodies, including anti-Beta Actin (20536-1-AP, Proteintech, China), anti-α-SMA (14395-1-AP, Proteintech, China), anti-CD31 (11265-1-AP, Proteintech, China), anti-E-cadherin (20874-1-AP, Proteintech, China), anti-Vimentin (10366-1-AP, Proteintech, China), anti-ZEB1 (21544-1-AP, Proteintech, China), anti-HIF1α (20960-1-AP, Proteintech, China), anti-HIF1α-OH402 (ab72775, Abcam, USA), anti-HIF1α-OH564 (#3434, CST, USA), anti-PHD1 (12984-1-AP, Proteintech, China), anti-PHD2 (19886-1AP, Proteintech, China), and anti-PHD3 (18325-1-AP, Proteintech, China) were used. Secondary antibodies, consisting of goat anti-mouse and anti-rabbit antibodies conjugated with horseradish peroxidase (Proteintech, China), were utilized, and the blots were detected utilizing enhanced chemiluminescence (ECL) (P10300, NCM, China). Quantitative analysis of western blotting was performed using ImageJ.

### RNA extraction and real-time polymerase chain reaction (PCR) assay

RNA isolation was performed using the FastPure Cell/Tissue Total RNA Isolation Kit V2 (RC112-01, Vazyme, China) according to the manufacturer's protocol. Complementary DNA (cDNA) was synthesized from the isolated RNA using the HiScript III All-in-one RT SuperMix Perfect for qPCR Kit (R333-01, Vazyme, China). Real-time PCR was carried out on the synthesized cDNA using the Taq Pro Universal SYBR qPCR Master Mix Kit (Q712-02, Vazyme, China) in real-time PCR system (ABI7500, Applied Biosystems, USA). The mRNA levels of target genes were quantified using the 2^−ΔΔCT^ method. Please refer to Additional file 1: Table S1 for the specific primers utilized in the analysis.

### Cell viability, invasion and migration assays

Cell viability was determined using the cell counting-8 (CCK8) kit (Dojindo, Kumamoto, Japan) according to the manufacturer's instructions. HO8910 and A2780 cells were seeded individually at a density of 2.5 × 10^3^ cells per well in 96-well plates. After the incubation period, 10 µl of the CCK8 reagent solution were added to each well and incubated at 37 °C for 2 h. Subsequently, the optical density at a wavelength of 450 nm was measured to assess cell viability. Cell invasion assays were conducted using transwell chambers pre-coated with diluted BD Matrigel (1:15, BD Biosciences, USA) in 24-well plates. A cell suspension containing 4 × 10^4^ cells was seeded onto the upper chambers. The lower chambers were filled with 500 μl of different mediums (serum-free). Following a 48-h incubation period, non-invading cells and Matrigel on the upper chambers were carefully removed. The cells were then fixed with 4% paraformaldehyde and stained with a 0.1% crystal violet solution. For cell migration, cells at 80–100% confluence were cultured in a 6-well plate and a scratch was created using a sterilized 200 μL pipette tip. Different groups of conditioned mediums (serum-free) were then added and cultured for 24 h in a 37 °C incubator.

### Enzyme-linked immunosorbent assay (ELISA)

FGF7 levels in the supernatant of different groups were measured using a human FGF7 ELISA kit (R&D Systems, USA) following the manufacturer's instructions. The absorbance intensity of each sample was then measured at a wavelength of 450 nm using an automated microplate reader.

### Cell transfection, lentivirus transduction and stable cell lines

Short hairpin RNA against human ZEB1 (sh-ZEB1), short hairpin RNA against human HIF-1α (sh-HIF-1α), and negative control shRNA (sh-NC) were obtained from GenePharma. Cells were seeded in six-well culture plates one day prior to transfection and were transiently transfected using Lipofectamine 3000 (Invitrogen, USA) following the manufacturer's instructions. To generate stable cell lines, puromycin at a concentration of 2 mg/ml was added to screen the transfected cells. In addition, HO8910 and A2780 cells were transfected with small interfering RNA (HIPPOBIO, China) to knock down FGF7. After 48 h of transfection, the efficiency of knockdown and overexpression was validated through western blotting and quantitative real-time PCR.

### Animal experiments

All animal experiments adhered strictly to the guidelines set by the Medical Research Animal Ethics Committee of Southeast University. The animal experiments comprised the utilization of six-week-old female nude mice (BALB/c-nu) with five mice per group, and were conducted following established protocols [16]. For the tumor xenograft experiments, a cell suspension mix containing 1.6 × 10^6^ HO8910 cells and 4 × 10^5^ CAFs or NFs cells (in a 4:1 ratio) was subcutaneously injected into the animals. Beginning on the second day post-injection, the animals received intraperitoneal injections of either FGF7 neutralizing antibodies or IgG (50 mg/kg). The growth of the tumors was monitored and recorded every five days. On day 31 after cell injection, all mice were humanely euthanized. Visible tumors were recorded, and the tumor tissues were excised, weighed, and then embedded in paraffin for subsequent analysis. Tumor volume was calculated using the formula: Tumor volume (mm^3^) = (d^2^ × D) / 2, where D and d represent the longest and shortest diameters of the tumor, respectively.

### Immunohistochemistry (IHC) and Immunofluorescence staining

The 4 mm paraffin-embedded tissue sections were subjected to immunohistochemical staining (IHC). IHC staining of subcutaneous graft tumors and human tissues was performed by a reputable commercial entity (Servicebio, China). For immunofluorescence, two formalin-fixed paraffin-embedded (FFPE) tissue blocks (FIGO I and FIGO III tumors) were selected from Zhongda Hospital, Southeast University. Immunofluorescence staining was also conducted by the aforementioned commercial entity. The antibodies used in the immunofluorescence panels included Vimentin (Servicebio, GB121308), FOXP3 (Servicebio, GB112325) and α-SMA (Servicebio, GB13044). Lastly, DAPI was utilized as a counterstain for nuclear labeling. Detailed methods can be found in a previously published work by the same company [3, 16]. Stained slides underwent analysis using a NanoZoomer S360 slide scanner (Hamamatsu Photonics, France). For tumor area assessment, the IHC staining were quantified using Quant Center software. Staining intensities were classified into weak, moderate, or strong categories. The proportion of cells within each intensity category was calculated, and the IHC score was derived using the formula: IHC score = (percentage of weakly stained cells × 1) + (percentage of moderately stained cells × 2) + (percentage of strongly stained cells × 3).

### Cleavage under targets and release using nuclease assay (CUT&RUN)

To investigate the binding of HIF-1α to the promoter region of ZEB1, we employed the CUT&RUN technique. The CUT&RUN assay was conducted using the CUT & RUN Assay Kit (HD101-01, Vazyme, China) according to the manufacturer's instructions [17]. Cells were treated with various conditioned media for 48 h. After centrifugation and resuspension, 1 × 10^5^ cells per sample were utilized for the experiments. Briefly, cells were incubated with pre-treated ConA Beads Pro for 10 min, followed by immunoprecipitation with the corresponding primary antibody for 2 h at room temperature. Subsequently, pG-MNase, bound to the cells, was activated by CaCl_2_. Following a 30-min abortive termination step of the reaction system at 37 °C, the reaction system was centrifuged to collect the supernatant containing chromatin-rich products. An equal number of cells were employed in each group, and 10 pg spikes were added to each sample for calibration. The data were presented as 2 − ^△△CT^ values, normalized to the control group. For specific primers used in the analysis, please refer to Additional file 1: Table S1.

### Co-immunoprecipitation (Co-IP) assay

Cells underwent treatment with lysis buffer containing complete protease inhibitors. These cells were then incubated with 50 μl of Protein G Magnetic Beads (#70024, Cell Signaling Technology) previously conjugated with specific antibodies. This incubation occurred at 4 °C overnight. Subsequently, the beads were washed five times and centrifuged at 13,000 rpm to isolate the immunoprecipitates, which were then analyzed by Western blotting. The primary antibodies used in this process included anti-Ubiquitin (#3936, CST) and anti-pVHL (#68547, CST).

### Bioinformatics analysis of single cell transcriptome

The scRNA-seq data used in this study were obtained from Women's Hospital, Zhejiang University School of Medicine, and were collected with appropriate ethical approval. The raw data have been deposited in the Gene Expression Omnibus (GEO) database [18] under the accession code "GSE184880". scRNA-seq experiments were performed by experimental personnel in the laboratory of Novel Bio Co, Ltd. In brief, we retained a total of five samples from the original dataset, which included I-stage samples (GSM5599227, GSM5599228 and GSM5599231) localized to the surface of the ovary or fallopian tube, as well as III-stage samples (GSM5599225 and GSM5599230) that exhibited peritoneal and retroperitoneal lymph node metastasis. The samples were procured exclusively from primary tumors. Briefly, the original quality-controlled files were downloaded from the GEO database and the cells were dimensionalized, clustered, and annotated using the protocol from the original study [19]. It's worth noting that we utilized the Harmony algorithm [20] to mitigate batch effects among the samples. Subsequently, we carried out comprehensive cell annotation, categorizing the entire cell population into eight distinct types: T cells, epithelial cells, endothelial cells, CAFs, myofibroblasts, NK cells, B cells or plasma cells, and monocytes. To assess the signaling inputs and outputs between CAFs and other cells, we employed the "CellChat" package [21]. We used the set of EMT-related genes from previous publications [3] to score epithelial cell using the “AddModuleScore” algorithm.

### Bioinformatics analysis of bulk transcriptome

In our study, we incorporated samples that possessed comprehensive survival information, including survival time, while ensuring that the overall survival (OS) was greater than 30 days. Specifically, we focused on primary serous cases to ensure the homogeneity of the sample population. After excluding patients with samples sequenced multiple times from the same patient, we downloaded RNA sequencing (RNA-seq) in each kilobase of transcript per million mapped reads (FPKM) format from both the Cancer Genome Atlas (TCGA) database [22] and the International Cancer Genome Consortium (ICGC) database. The original data was logarithmically transformed using log2 [(TPM) + 1]. We integrated the ICGA-OV cohort (n = 111) and TCGA-OV cohort (n = 364) to define the RNA-seq dataset (n = 475). Data from the Gene Expression Omnibus (GEO) database was obtained for the GPL570 platform (n = 472; including GSE19829, GSE18520, GSE9891, GSE26193, GSE30161, and GSE63885), the GPL7759 platform (n = 413, including GSE13876), the GPL96 platform (n = 395, including GSE3149, GSE23554, GSE276712, and GSE14764), the GPL14951 platform (n = 273, including GSE140082), the GPL2986 platform (n = 193, including GSE49997), and the GPL6480 platform (n = 405, including GSE17260, GSE32063, and GSE32062). The "sva" package [23] was utilized to eliminate batch effects across the various datasets. For specific information of datasets used in the analysis, please refer to Additional file 1: Table S2.

CAFs scores were computed using three distinct methods: the Estimate the Proportion of Immune and Cancer cells (EPIC) algorithm, the xCell algorithm, and the microenvironment cell populations-counter (MCP-counter) algorithm. To facilitate these computations, we utilized the "IOBR" package [24]. For different transcriptomic platforms and different immune infiltration algorithms, it was not possible to generate consistently inter-comparable CAFs scores. To stratify patients into optimal groups (High CAFs or Low CAFs) for survival analysis, we applied the “surv_cutpoint” function from the “survminer” package, enabling optimal cut-off.

Additionally, the identification of hub genes significantly associated with CAFs scores was performed using the "WGCNA" package [25]. Initially, the expression profiles demonstrating the top 25% variance in both the GPL570 cohort and the RNA-seq cohort were selected as input data. Subsequently, following our established workflow [3], we correlated the phenotypes and modules by identifying appropriate soft thresholds and integrating gene modules that exhibited similar characteristics.

The machine learning models employed in our study included Lasso, CoxBoost, RSF (Random Survival Forest), StepCox (both forward and backward steps), GBM (Gradient Boosting Machine), Survival-SVM (Support Vector Machine), SuperPC (Super Principal Components), ridge regression, plsRcox (Partial Least Squares—Cox), and enet (Elastic Net). Lasso is a method that utilizes regularization to select relevant variables by imposing a penalty on the absolute values of coefficients [26]. CoxBoost is a boosting algorithm specifically designed for survival analysis, capable of handling high-dimensional data [27]. RSF is a random forest-based approach tailored for survival analysis, which uses an ensemble of decision trees to predict survival outcomes [28]. StepCox involves stepwise selection of variables based on their significance in Cox proportional hazards models. Additionally, GBM is an ensemble learning technique that combines weak prediction models, often decision trees, to build a strong predictive model [29]. Survival-SVM uses support vector machines to predict survival outcomes by finding an optimal hyperplane that maximally separates the data [30]. SuperPC is a dimension reduction method that combines several principal component analyses to extract key features [31]. Ridge regression is a linear regression method that introduces a penalty term to handle multicollinearity [32]. PLSRcox is a method that combines partial least squares regression with Cox proportional hazards models [33]. Lastly, enet is a regularization approach that combines L1 (Lasso) and L2 (ridge regression) penalties to achieve variable selection and parameter estimation simultaneously [34]. We utilized an RNA-seq cohort and validated it against multiple platforms, namely GPL570, GPL7759, GPL96, GPL6480, and GPL14951. Following established workflow [35, 36], each dataset underwent separate standardization through mean/variance normalization. In the RNA-seq dataset, variable selection was performed using Lasso, CoxBoost, RSF, StepCox (both), and StepCox (backward) methods. Ten algorithms, including lasso, RSF, GBM, Survival-SVM, SuperPC, ridge regression, plsRcox, CoxBoost, StepCox, and enet, were subsequently employed to create a combined model integrating these machine learning algorithms. To select the optimal prognostic model, the average C-index across the datasets was utilized.

The “clusterProfiler” package was utilized to perform GO (Gene Ontology) and KEGG (Kyoto Encyclopedia of Genes and Genomes) enrichment analyses [37]. The process involved input a gene list and converting to the Entrez id to detect significantly GO terms or KEGG pathways. Benjamini–Hochberg procedure was applied to control for false discovery rates (p.adj < 0.05). To perform GSVA (Gene Set Variation Analysis) [38] for the hallmark gene concentrated hypoxia pathway, we utilized “GSVA” package. The process involved inputting gene expression data and the predefined hallmark gene set. GSVA was then applied to calculate pathway activity scores for each sample, reflecting the enrichment of the hypoxia pathway. This analysis allows for the quantification of pathway activity across samples and facilitates the identification of potential associations with hypoxia-related functions.

### Statistical analysis

Statistical analyses were performed to assess the significance of observed differences and correlations in the study. All data were expressed as mean ± SD (standard deviation). Student’s t-test was used to analyze significant differences between two groups, while one-way or two-way ANOVA followed by post-hoc tests were employed for multiple group comparisons. To evaluate the impact of risk factors on survival outcomes, Cox regression models were utilized. This allowed for the determination of hazard ratios (HR) and calculation of corresponding 95% confidence intervals (CI). Kaplan–Meier (K-M) survival analysis was performed to estimate and visualize the survival probabilities over time, with log-rank tests used to assess the statistical significance of differences between survival curves. Pearson correlation analysis was conducted to explore the relationships between variables. This analysis measured the strength and direction of linear associations, providing correlation coefficients (r) along with p-values. Statistical analysis and scientific graphing were performed using R Studio (version 4.1.1) and GraphPad Prism (version 9.0). A significance level of P < 0.05 was considered statistically significant.

## Results

### Multiple cohorts and algorithms confirm poor prognosis association of CAFs

**Fig. 1:**
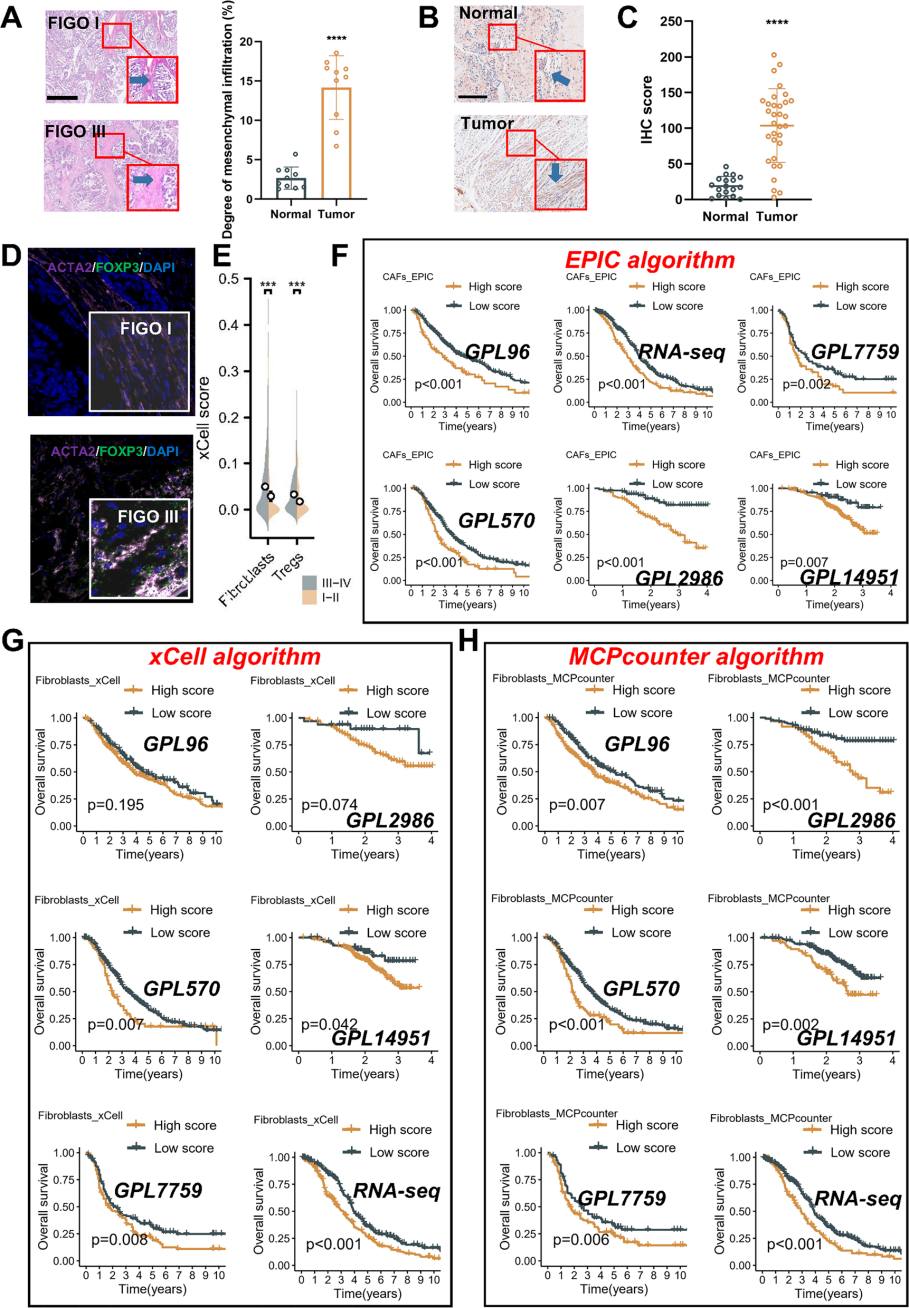
Confirmation of the poor prognosis association of CAFs using multiple datasets and algorithms. **A** Hematoxylin and eosin (H&E) staining images displaying OC tissues at different stages. Histogram of the percentage of matrix in different samples. FIGO I sampels (n = 10), FIGO III sampels (n = 10), Scale bar: 100 μm. **B** Immunohistochemical staining images showing the expression of α-smooth muscle actin (α-SMA) in different tissues. Scale bar: 100 μm. **C** Histogram of ACTA2 score in different samples.Normal sampels (n = 18), OC sampels (n = 32). **D** Representative morphological images obtained through multiple fluorescence staining techniques, depicting tissues from FIGO stage I (upper) and FIGO stage III (lower) patients. The purple color represents α-SMA positivity, the red color indicates FOXP3 positivity, and the blue color indicates DAPI staining for nuclear identification. Scale bar: 100 μm. **E** Differential distribution of cells (CAFs and Tregs) across samples in the TCGA-OV cohort (xcell algorithm). **F** Kaplan–Meier plot illustrating survival analysis based on CAFs scores in different cohorts, utilizing the EPIC algorithm. **G** Kaplan–Meier plot showcasing survival analysis based on CAFs scores in different cohorts, utilizing the xCell algorithm. **H** Kaplan–Meier plot demonstrating survival analysis based on CAFs scores in different cohorts, employing the MCPcounter algorithm. Note: GPL570 cohort (n = 472; GSE19829, GSE18520, GSE9891, GSE26193, GSE30161, and GSE63885), GPL7759 cohort (n = 413, GSE13876), GPL96 cohort (n = 395, GSE3149, GSE23554, GSE276712, and GSE14764), the GPL14951 cohort (n = 273, GSE140082), the GPL2986 cohort(n = 193, GSE49997), and the RNA-seq cohort (n = 475, TCGA-OV and ICGC-OV)

The representative hematoxylin and eosin (H&E) images demonstrate enhanced matrix accumulation in FIGO III samples (Fig. 1A). Additionally, IHC score of ACTA2 (α-SMA), reveals stronger staining intensity in OC tissues compared to normal tissues (Fig. 1B, C). Representative fluorescent images corroborated that regions with high CAFs density also exhibited increased recruitment of Tregs (Fig. 1D), contributing to immune tolerance [39]. Concurrently, quantification of CAFs and Tregs in various samples using the xCell algorithm revealed higher score of both cell types in patients at FIGO III-IV stages (Fig. 1E). The references have consistently reported a strong correlation between immunosuppression and tumor prognosis [39]. In light of this, we used multiple datasets and various algorithms to establish robust evidence supporting the prognostic value of CAFs. To this end, we utilized six meta cohorts (RNA-seq, GPL570, GPL2986, GPL96, GPL7759, GPL14951) and three algorithms (EPIC, MCPcounter, xCell) to verify our findings (Additional file 1: Table S2). Encouragingly, our results consistently demonstrated a significant association between higher CAFs infiltration and poor OS in patients across all examined datasets, with only some exceptions noted in the xCell algorithm datasets (Fig. 1F–H).

Taken together, the findings from multiple datasets and algorithms provide compelling evidence that CAFs are associated with poor prognosis in OC.

### WGNCA algorithm identifies key genes derived from CAFs

We employed Weighted Gene Co-expression Network Analysis (WGCNA) to identify key genes originating from CAFs in OC. Unlike methods that solely focus on differentially expressed genes, WGCNA utilizes the entire dataset, transforming the extensive array of gene-to-phenotype associations into a more manageable number of relevant sets. Moreover, this approach effectively addresses the issue of adjusting for multiple hypothesis testing in differential expression analysis [25]. The RNA-seq cohort and the GPL570 cohort, which had larger sample sizes, were analyzed using WGCNA. We initially confirmed the absence of significant outlier samples in each cohort and calculated the correlation matrix between genes using the average linkage matrix and Pearson correlation method. The formula amn =|cmn|β was used to transform the correlation matrix into the adjacency matrix. For both cohorts, a soft threshold power (β) of 3 was determined to be the optimal choice (Additional file 1: Fig. S1A). Following the steps of WGCNA, a gene network was constructed after hierarchical clustering. The dynamic pruning tree method merged similar genes into gene modules, with a minimum of 50 genes per module. Ultimately, eight modules were identified in the RNA-seq cohort, while nine modules were discovered in the GPL570 cohort (Additional file 1: Fig. S1B).

We then examined the correlation between CAFs (clinical trait) and the modules, calculating correlation coefficients and p-value (Additional file 1: Fig. S1C). To ensure the robustness of the trait, we employed two algorithms, EPIC and MCPcounter, which yielded consistent results in survival analysis in above section. Notably, the blue modules in both cohorts exhibited a strong correlation with CAF levels (r > 0.8, p < 0.05). Furthermore, when assessing the association between the proportion of blue modules and CAFs, both gene significance (GS) and module membership (MM) correlations exceeded 0.9 (Additional file 1: Fig. S1D). This indicates that all genes within the blue modules were specifically expressed by CAFs in OC and remained unaffected by the influence of other cell types. MM measures the degree of association between each gene and a given co-expression network module, while GS measures the degree of association between each gene and an external trait (CAFs) [25]. Genes with high MM and high GS values may have important biological functions in the study.

**Fig. 2:**
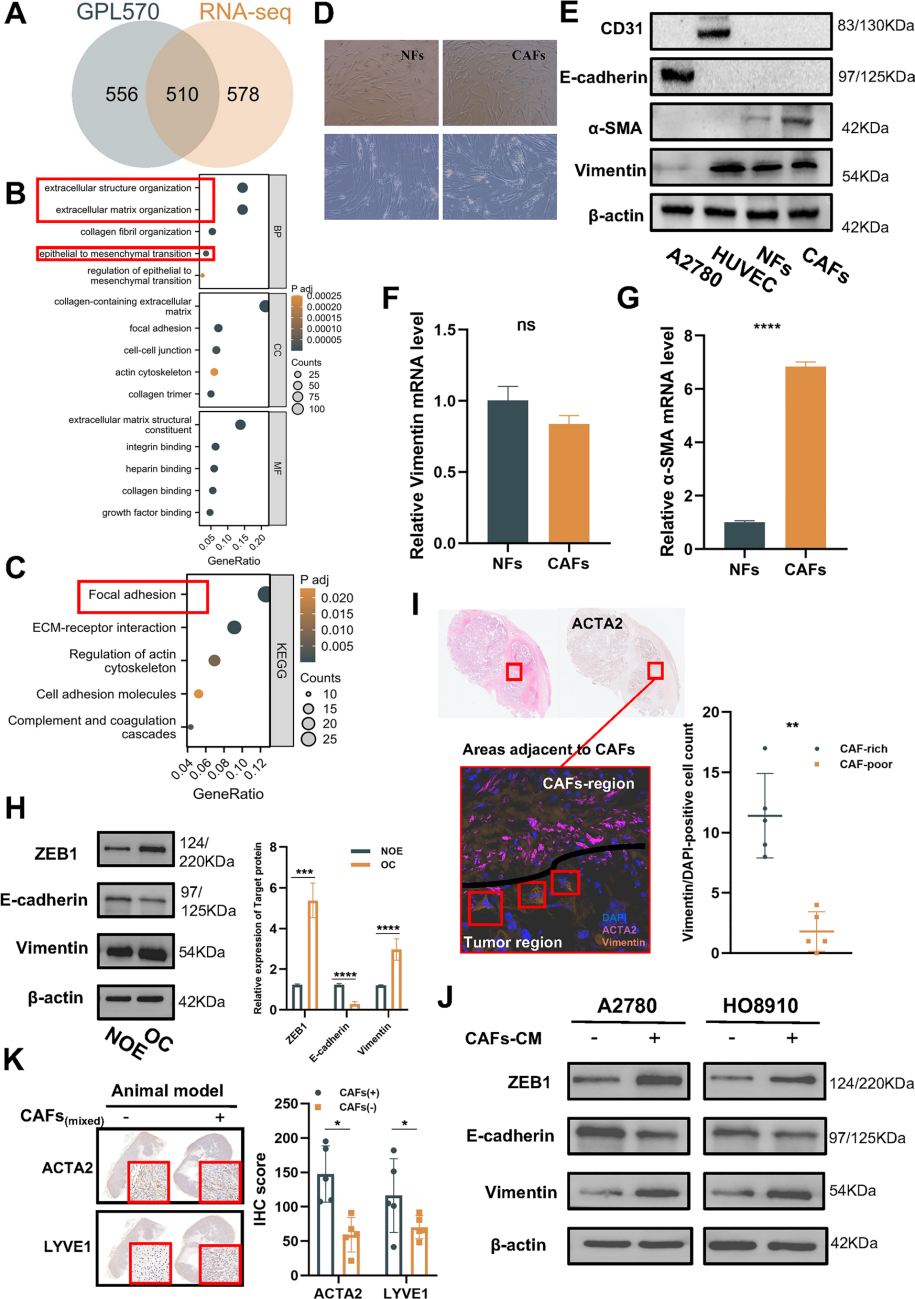
Screening of prognostic-related genes derived from CAFs. **A** Venn diagram illustrating the overlap of 510 key genes identified in both the RNA-seq cohort and GPL570 cohort. **B** Gene Ontology (GO) enrichment analysis demonstrating the functional significance of the identified key genes derived from CAFs. The bubbles represent different biological processes (BP), cell localizations (CC), and molecular functions (MF), with each bubble's size corresponding to the number of enriched genes in that specific category. **C** Kyoto Encyclopedia of Genes and Genomes (KEGG) enrichment analysis further validating the functional relevance of the key genes derived from CAFs. The size of each bubble represents the number of enriched genes associated with specific pathways. **D** The upper image (100 × magnification) shows 60–70% confluent cells, while the lower image shows purified CAFs or NFs (200 × magnification). **E** Western blotting analysis presenting the expression of representative markers (α-SMA, CD31, E-cadherin, and Vimentin). **F** RT-qPCR analysis presenting the expression of the representative marker Vimentin. **G** RT-qPCR analysis presenting the expression of the representative marker α-SMA. **H** Western blotting analysis of the expression of epithelial-mesenchymal transition (EMT) markers in different tissues. **I** Representative morphological images obtained through multiple fluorescence, IHC, and H&E staining techniques, depicting tissues from OC tissues. The purple color represents α-SMA positivity, the orange color indicates Vimentin positivity, and the blue color indicates DAPI staining for nuclear identification. Scale bar: 100 μm. **J** Western blotting analysis of the expression of EMT markers in A2780 and HO8910 cells pretreated with CAFs-CM. **K** IHC analysis of the expression of ACTA2 and LYVE1 in tumor tissues from vivo assays (10 × magnification). Histogram of the IHC score in different samples. CAFs positive samples (n = 5), CAFs negative sampels (n = 5). Results are presented as the mean ± SD of three independent experiments. ****P < 0.0001, ns not significant

Finally, we successfully identified a total of 1088 and 1066 key genes in the RNA-seq cohort and GPL570 cohort, respectively, with 510 genes common to both cohorts (Fig. 2A).

### Strong correlation between EMT and CAFs

Utilizing enrichment analysis, we proceeded to investigate the functional relevance of 510 key genes derived from CAFs (Fig. 2B, C). Notably, we observed a strong correlation between these genes and important biological processes related to the extracellular matrix (ECM) and epithelial-mesenchymal transition (EMT). To investigate the association between key genes derived from CAFs and EMT, a series of experiments were conducted. CAFs were isolated and their supernatants collected to assess their impact on EMT. Subsequently, CAFs and NFs were isolated and cultured from OC and normal ovarian epithelial tissues, respectively (Fig. 2D). The characterization of the isolated cells was determined by analyzing α-SMA and Vimentin (mesenchymal cell marker) expression. Notably, the expression of E-cadherin (epithelial cell marker) and CD31 (endothelial cell marker) was found to be negative, indicating the absence of epithelial or endothelial cell contamination (Fig. 2E–G). as two positive control cell lines exhibited significant expression of CD31 and E-cadherin, respectively. Moreover, the significant expression of CD31 and E-cadherin in two positive control cell lines (A2780 and HUVEC) further validates the effectiveness and accuracy of our characterization.

Interestingly, Western blot experiments on normal ovarian epithelium (NOE) and OC tissue revealed significant changes in EMT-related markers, with increased expression of Vimentin and ZEB1 and decreased expression of E-cadherin (Fig. 2H). To investigate whether these changes were related to CAFs, we performed immunofluorescence, H&E staining, and IHC on human OC FFPE tissue. We compared co-layered H&E sections and ACTA-stained IHC sections to identify CAF-rich regions as well as CAF-poor regions in their mIHC (No staining and weak staining were defined as CAFs-poor regions; moderate staining and strong staining were defined as CAFs-rich regions). We only compared Vimentin/DAPI double-positive cells around CAFs. Representative areas showed that more double-positive cells were detected in areas with significant aggregation of CAFs, while double-positive cells were significantly reduced in areas with CAFs-poor (Fig. 2I). Consistently, Western blot analysis demonstrated alterations in EMT-related markers in cells treated with CAFs-CM (Fig. 2J), mirroring findings observed in advanced patients. Finally, animal experiments (inoculation at the footpad site) using mixed CAFs revealed enhanced expression of ACTA2 and the lymphatic vessel marker LYVE1 in the resulting tumors (Fig. 2K), providing potential evidence for CAFs promoting metastasis. Based on these results, we hypothesize that CAFs, which are rich in OC microenvironments, can affect adjacent cancer cells via paracrine secretion, leading to up-regulation of the EMT pathway.

Taken together, the results of this study indicate a strong correlation between key genes derived from CAFs and biological processes associated with the ECM and EMT, suggesting their involvement in tumor progression. The experimental findings demonstrate that these CAFs-derived genes can induce EMT in OC cells and promote progression, providing additional evidence for their role in driving OC development.

### Machine learning identifies FGF7 as most key gene derived from CAFs

**Fig. 3:**
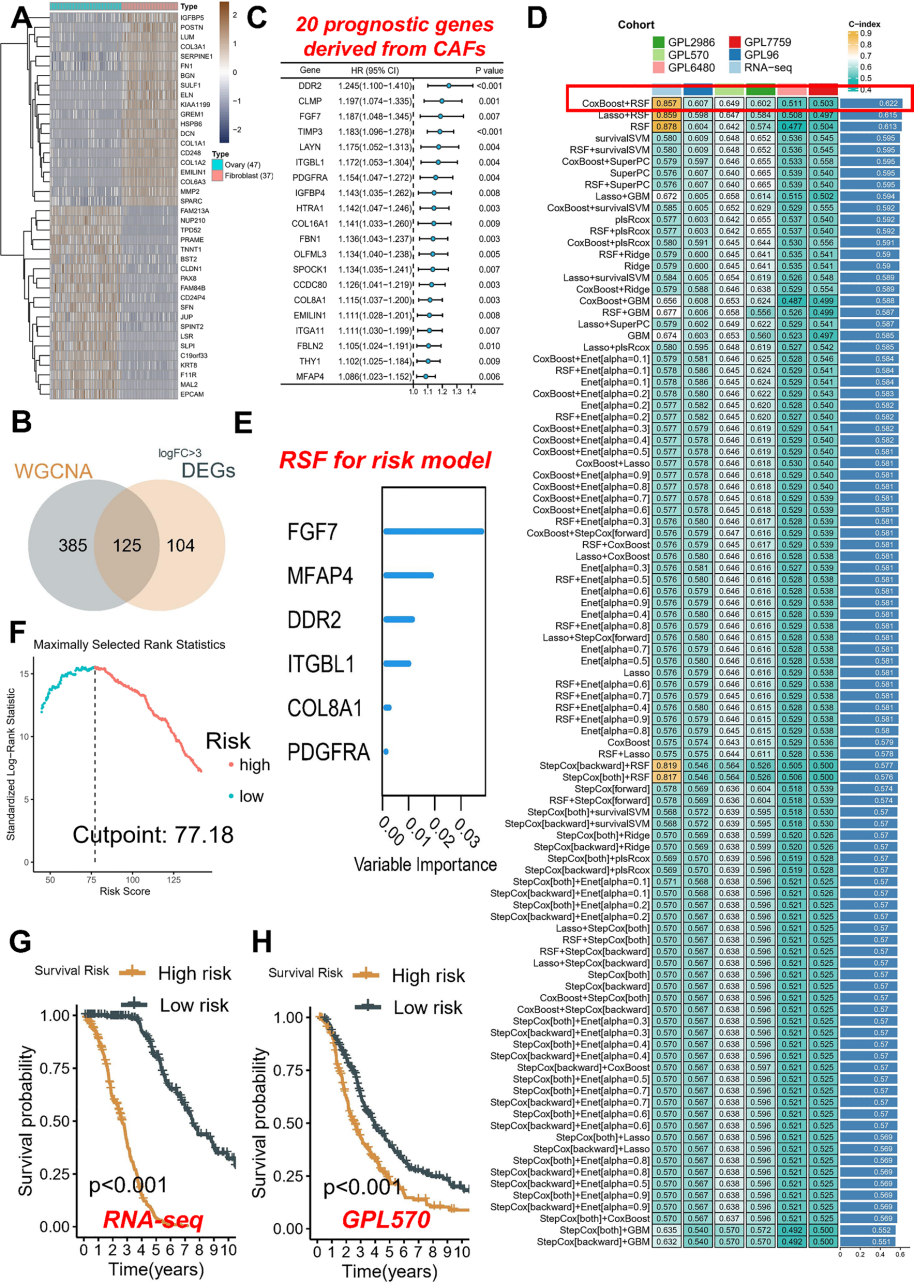
Machine learning screening of key molecules. **A** Heat map visualizing the differential expression pattern of genes in RNA-seq data obtained from ovarian cancer (OC) cell lines and fibroblast cell lines sourced from the Cancer Cell Line Encyclopedia (CCLE) database. The heat map highlights the distinct gene expression profiles between OC and fibroblast cells. **B** Venn diagram demonstrating the intersection between the identified 229 differential genes and the previously established set of 510 key genes. **C** Forest plot presenting the results of univariate Cox regression analysis, which screened 20 genes derived from CAFs for their association with prognosis. The plot quantifies the hazard ratios and confidence intervals for each molecule. **D** Heat map illustrating the C-index values of 99 combined algorithms in each cohort. The rightmost column represents the average C-index values of the six cohorts. **E** Ranking of the importance of six molecules in random forest tree models. **F** Determination of the optimal cutoff value to classify the entire patient population into high-risk and low-risk groups. **G** Kaplan–Meier survival analysis of the RNA-seq cohort. **H** Kaplan–Meier survival analysis of the GPL570 cohort

Because the data source for the WGCNA remains bulk transcriptome, we validated it using highly expressed genes from fibroblast cell lines (logFC > 3) in order to determine the specificity of the source of CAFs. Hence, we conducted a differential analysis utilizing RNA-seq data from 47 OC cell lines and 37 fibroblast cell lines available in the Cancer Cell Line Encyclopedia (CCLE) database (Fig. 3A). Excitingly, we discovered that the set of differential genes significantly overlapped with the previously mentioned 510 key genes, resulting in a total of 125 overlapping genes (Fig. 3B). To gain further insights into the prognostic implications of the identified genes, we performed univariate Cox regression analysis to screen 20 genes derived from CAFs associated with prognosis (Fig. 3C). Of particular interest, we found that all of the identified genes are associated with an increased risk of poor survival in patients with OC.

To deeply identify genes associated with CAFs among the previously mentioned 20 prognostic genes, we employed a comprehensive approach utilizing 99 machine learning algorithms. This allowed us to effectively screen for key genes with significant implications. Subsequently, these key genes were utilized to devise a novel risk score, enabling accurate assessment of the prognosis for individual patients with OC. Our findings uncovered that the implementation of coxboost in conjunction with RSF yielded the most promising results, as demonstrated by the highest average C-index (0.622) across all six cohorts (Additional file 1: Table S3, Fig. 3D). As the boosting steps increase, the coefficients associated with each variable undergo modifications, reflecting their contribution to the predictive power of the coxboosting model. The RSF model was used among the filtered variables. At the top of the list is FGF7, indicating its prominent role as the most influential variable in our study. Following FGF7, we identified MFAP4, DDR2, ITGBL1, COL8A1, and PDGFRA as key variables deserving further attention (Fig. 3E). We sought to evaluate the predictive power of a newly generated risk score derived from six CAFs-derived genes. This allowed us to determine an optimal cut-off value for risk stratification, which was found to be 77.18 (Fig. 3F). Subsequently, this risk score was employed to categorize patients into high-risk and low-risk groups in all six cohorts under investigation. Consistent with our expectations, we observed that the high-risk group exhibited significantly shorter survival times compared to the low-risk group in RNA-seq cohort (Fig. 3G) and GPL570 cohort (Fig. 3H). More importantly, other validation cohorts also demonstrated consistent survival validation results (Additional file 1: Fig. S2A-D).

These findings underscore the robustness and efficacy of our machine learning model in accurately identifying patients at high risk for poor survival outcomes.

### FGF7 in CAFs-CM regulates progression of ovarian cancer

To further explore the clinical implications of FGF7 expression, we performed correlation analyses using the TCGA cohort. Remarkably, our results demonstrated that patients with high FGF7 expression were frequently diagnosed at advanced stages of the disease. Additionally, this group exhibited a higher incidence of vascular invasion, lymphovascular invasion, and residual tumor presence following surgical intervention (Table 1). To elucidate the impact of CAFs derived FGF7 on the biological function of OC, we conducted a series of experiments using OC cell lines (HO8910 and A2780) cultured with various conditioned media (CM). Results from the wound healing assay indicated that both HO8910 and A2780 cells treated with CAFs-CM for 48 h exhibited enhanced migration capacity (Additional file 1: Fig. S3A). Moreover, the transwell assay demonstrated a significant increase in invasion potential for both cell lines when exposed to CAFs-CM compared to normal fibroblasts-conditioned media (NFs-CM) or the negative control (Additional file 1: Fig. S3B). Furthermore, the CCK8 assay revealed that both CAFs-CM and NFs-CM promoted the proliferation of OC cells, with CAFs-CM displaying a more pronounced effect (Additional file 1: Fig. S3C, D). To establish the role of CAFs-derived FGF7 and exclude the influence of tumor cells themselves, we employed siRNA-2 to knock down FGF7 expression specifically in A2780 and HO8910 (Additional file 1: Fig. S3E, F). Interestingly, silencing FGF7 in OC cells alone did not inhibit cell proliferation (Additional file 1: Fig. S3G, H), migration (Additional file 1: Fig. S3I) and invasion (Additional file 1: Fig. S3J).

**Table 1:** Clinical correlation analysis of FGF7 in the TCGA-OV cohort

Characteristics	Low-FGF7	High-FGF7	P-value
Age, n (%)			0.715
< = 60	106 (27.8%)	103 (27%)	
> 60	84 (22%)	88 (23.1%)	
FIGO stage, n (%)			0.011
Stage I & Stage II	18 (4.8%)	6 (1.6%)	
Stage III & Stage IV	170 (45%)	184 (48.7%)	
Histologic grade, n (%)			0.828
G1&G2	22 (5.9%)	24 (6.5%)	
G3&G4	161 (43.4%)	164 (44.2%)	
Venous invasion, n (%)			0.012
No	27 (25.7%)	14 (13.3%)	
Yes	26 (24.8%)	38 (36.2%)	
Lymphatic invasion, n (%)			0.030
No	31 (20.8%)	17 (11.4%)	
Yes	46 (30.9%)	55 (36.9%)	
Tumor residual, n (%)			< 0.001
No	48 (14.2%)	20 (5.9%)	
Yes	120 (35.6%)	149 (44.2%)	

**Fig. 4:**
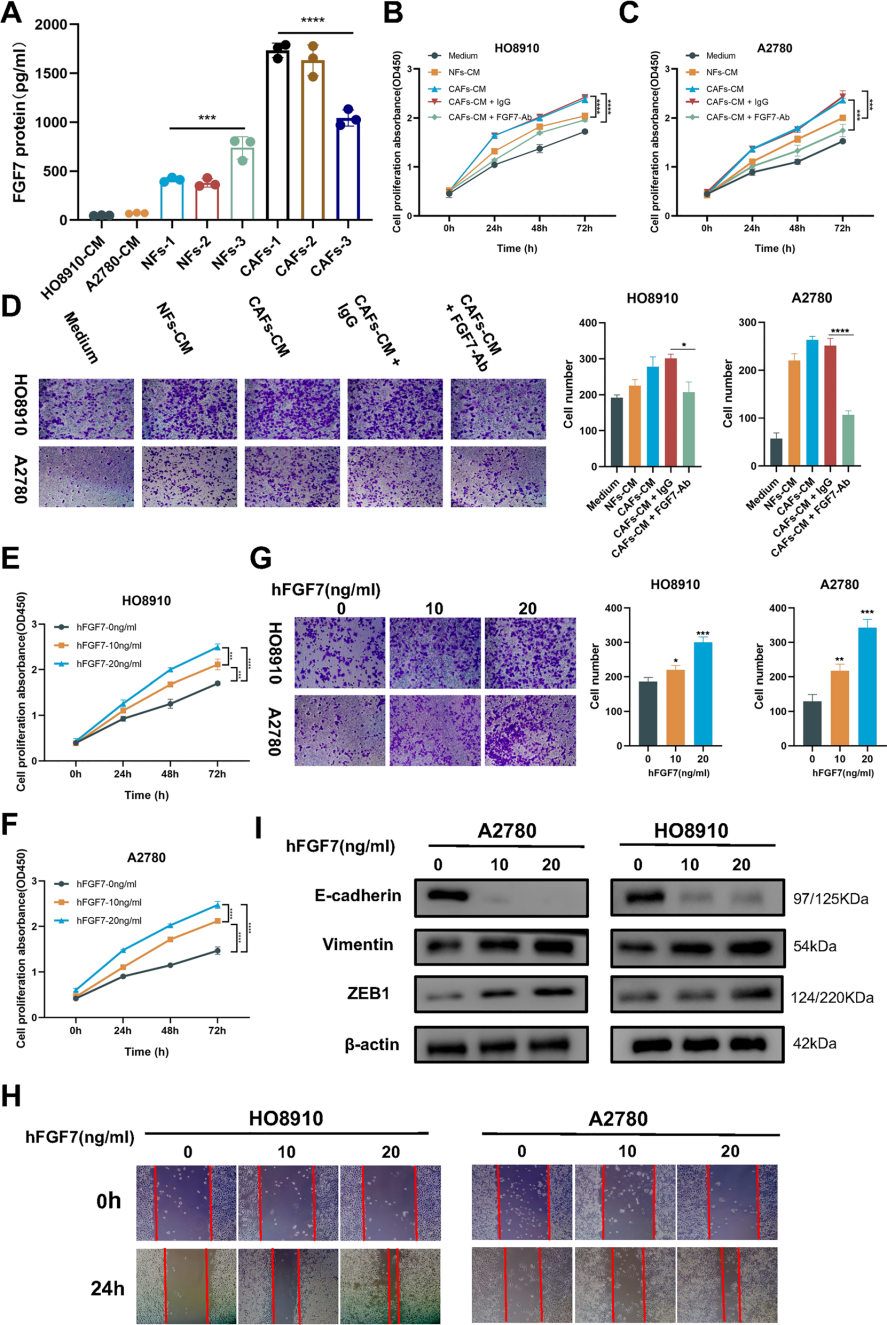
Role of CAFs-derived FGF7 in ovarian cancer progression. **A** Concentration of FGF7 in the medium of different cell types quantified using enzyme-linked immunosorbent assay (ELISA). **B** Cell viability of HO8910 cells measured by CCK8 assay after neutralizing FGF7 in CAFs-CM. **C** Cell viability of A2780 cells measured by CCK8 assay after neutralizing FGF7 in CAFs-CM. **D** Cell invasion ability evaluated by Transwell assay after 48 h in HO8910 and A2780 cells (200 × magnification). **E** Cell viability of HO8910 cells treated with different doses of hFGF7 measured by CCK8 assay. **F** Cell viability of A2780 cells treated with different doses of hFGF7 measured by CCK8 assay. **G** Cell invasion ability assessed by Transwell assay after 48 h in HO8910 and A2780 cells treated with different doses of hFGF7 (200 × magnification). **H** Wound healing assay used to measure cell migration ability after treatment with hFGF7 for 24 h (100 × magnification). **I** Expression of epithelial-mesenchymal transition (EMT) markers in ovarian cancer (OC) cells treated with different doses of hFGF7 examined by western blotting. Results are presented as the mean ± SD of three independent experiments. ****P < 0.0001, *P < 0.05

To validate these bioinformatic findings, we quantified the concentration of FGF7 in the medium of different cell using enzyme-linked immunosorbent assay (ELISA). As expected, FGF7 was significantly up-regulated in CAFs-CM, surpassing its expression levels in tumor cells and normal fibroblasts by several-fold (Fig. 4A). To further investigate the contribution of FGF7 derived from CAFs to OC progression, we cultured HO8910 and A2780 cells with CAFs-CM, NFs-CM, CAFs-CM combined with IgG (CAFs-CM + IgG), and CAFs-CM combined with FGF7-neutralizing antibody (CAFs-CM + FGF7-Ab). The CCK8 assay revealed that OC cells co-cultured with CAFs-CM exhibited greater viability compared to those co-cultured with NFs-CM. However, treatment with FGF7-Ab significantly inhibited cell proliferation (Fig. 4B, C). Similarly, the transwell assay demonstrated that both HO8910 and A2780 cells co-cultured with CAFs-CM displayed increased invasiveness, which was effectively mitigated by the addition of FGF7-Ab (Fig. 4D). Additionally, wound healing assays indicated that FGF7-Ab attenuated the migratory capacity induced by CAFs-CM (data not shown). We then sought to determine whether FGF7 derived from CAFs plays a pivotal role in driving OC progression. Treatment with various concentrations of human recombinant (hFGF7) led to increased proliferation (Fig. 4E, F), invasion (Fig. 4G), and migration (Fig. 4H) abilities in OC cells. Moreover, hFGF7 also induced EMT in OC cells by up-regulating Vimentin and ZEB1 expression while inhibiting E-cadherin expression, as indicative of a shift towards a more mesenchymal phenotype (Fig. 4I).

These findings suggest that CAFs and their secretion of FGF7 play a crucial role in driving OC progression and promoting a more aggressive phenotype.

### Single-cell sequencing analysis identifies significant interactions of FGF7 sources

**Fig. 5:**
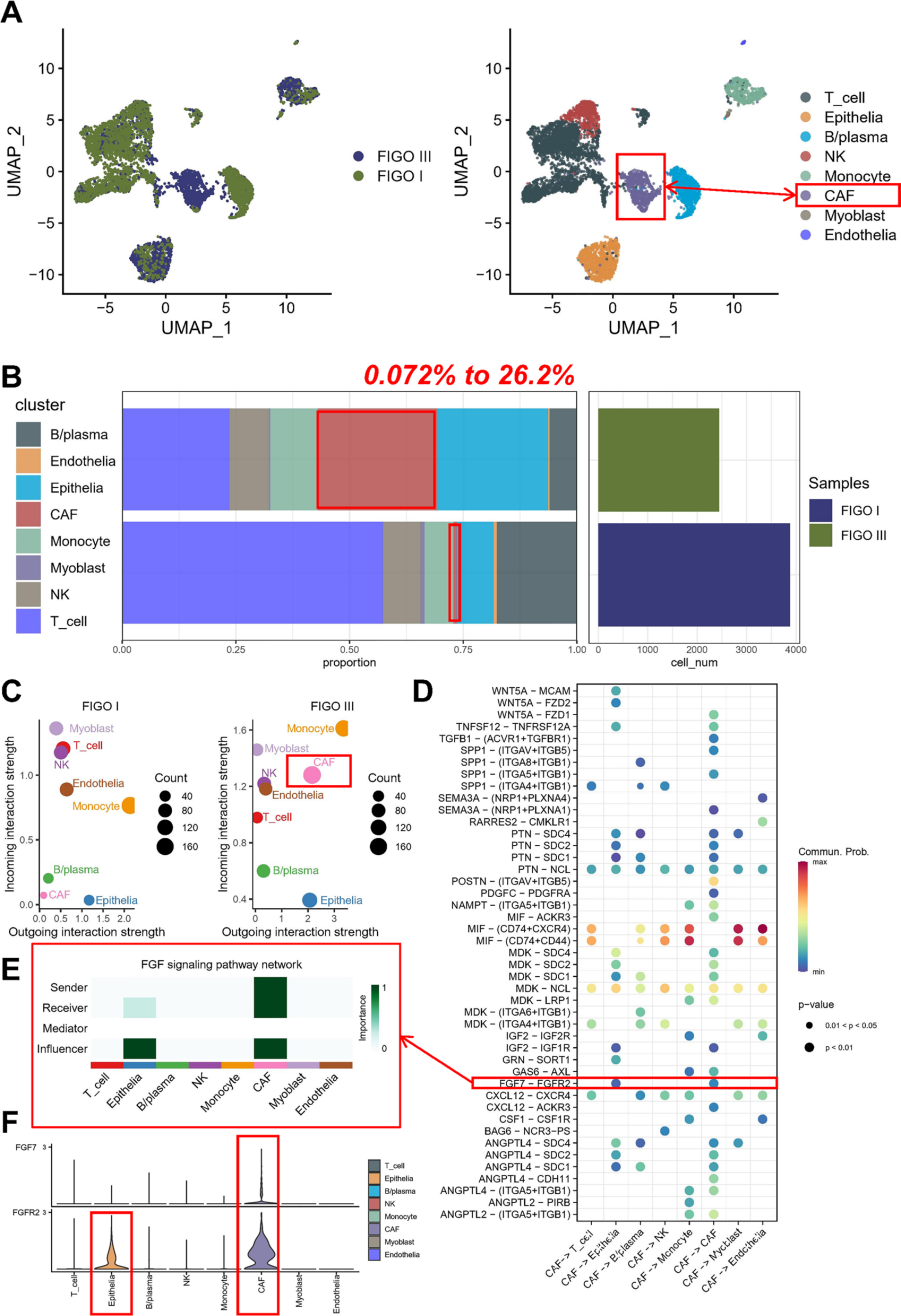
Characterization and communication of CAFs in single cell level. **A** Uniform Manifold Approximation and Projection (UMAP) plot illustrating major cell populations, with dots colored to represent different cell types: T cells, epithelial cells, endothelial cells, cancer-associated fibroblasts (CAFs), myofibroblasts, NK cells, B cells or plasma cells, and monocytes. **B** Bar plot presenting the fractions of different sample types relative to the total cell count per cell type in the samples analyzed. **C** Two-dimensional projections demonstrating input–output signal intensity of different cells in various samples. **D** Dot plot showcasing the ligand-receptor pairs involved in communication between CAFs and other cells. **E** Heatmap indicating the importance of different cells in FGF signaling pathway networks. **F** Violin plot displaying the expression levels of FGF7 and FGFR2 in different cell types

Given that FGF7 functions as a secreted protein, we aimed to investigate its interaction with tumor cells through its association with specific receptors. To achieve this, we conducted an analysis utilizing single-cell sequencing data. We comprehensively examined samples obtained from individuals with OC at various stages and subsequently classified the entire cells into eight categories: T cells, epithelial cells, endothelial cells, CAFs, myofibroblasts, NK cells, B cells or plasma cells, and monocytes (Fig. 5A). Remarkably, our analysis revealed a substantial increase in the proportion of CAFs as tumor progression occurred, with their representation escalating from a mere 0.072% to an astonishing 26.2% (Fig. 5B). Subsequently, we employed the “cellchat” package to infer the receptor-ligand interactions involving CAFs. The results demonstrated a significant enhancement in input–output signaling by CAFs following tumor progression (Fig. 5C). Moreover, our findings highlighted that CAFs-secreted FGF7 selectively bound to FGFR2 receptors located on the surface of epithelial cells (Fig. 5D). Notably, within this communication pathway, CAFs served as both senders and receivers, while the epithelial cells were solely influencers (Fig. 5E). It is worth mentioning that further analysis of gene expression patterns substantiated the elevated expression levels of FGFR2 in both CAFs and epithelial cells, with exclusive expression of FGF7 observed solely in CAFs (Fig. 5F).

It has been established that CAFs exhibit considerable heterogeneity in terms of their functional characteristics across various cancer types [7]. To explore this further within the context of OC, we conducted a comprehensive analysis to delineate distinct subtypes of CAFs present in OC dataset. We identified four distinct subtypes of CAFs within the OC samples (Additional file 1: Fig. S4A). Moreover, we observed a significant correlation between specific CAFs subtypes and different stages. Specifically, we found that Cluster-0 and Cluster-1 subtypes were more prevalent in FIGO III stage samples, while Cluster-2 and Cluster-3 subtypes were more commonly observed in FIGO I stage samples (Additional file 1: Fig. S4B). We highlighted the marker genes associated with each subtype: MMP11 for Cluster-0, NDRG1 for Cluster-1, C7 for Cluster-2, and RGS5 for Cluster-3 (Additional file 1: Fig. S4C). Notably, Cluster-0 demonstrated a strong correlation with the gene expression patterns of dCAF, iCAF, and pCAF, suggesting functional similarities among these subtypes. Conversely, Cluster-2 exhibited functional characteristics akin to those of iCAF-2, while Cluster-3 displayed similarities to myCAF. Intriguingly, Cluster-1 appeared to represent a unique CAF subtype that did not align with any of the previously identified pan-cancer classifications (Additional file 1: Fig. S4D).

Acknowledging the inherent heterogeneity of CAFs within the OC microenvironment and the challenges associated with isolating and categorizing each CAF subtype, we conducted additional investigations to ensure the robustness of our subsequent studies focused on the markers FGF7 and FGFR2. Initially, we examined the expression levels of ACTA2 (α-SMA), a commonly used marker for CAFs, across the different CAFs subtypes (Additional file 1: Fig. S4E). Surprisingly, no significant differences were observed in ACTA2 expression between the various CAFs subtypes. In order to further elucidate the significance of FGF7 (Additional file 1: Fig. S4F) and FGFR2 (Additional file 1: Fig. S4G) as potential markers for distinguishing between CAFs subtypes, we assessed their expression levels of CAFs. Interestingly, no significant differences were detected in the expression of FGF7 or FGFR2 across the different CAF subtypes. Consequently, to ensure the consistency and validity of our subsequent experiments, we proceeded with utilizing primary α-SMA-positive CAFs that were isolated from the OC samples. Interestingly, we further labeled the EMT score of the epithelial cells, which turned out to have more EMT-like cells in the FIGO stage III samples (Additional file 1: Fig. S4H).

**Fig. 6:**
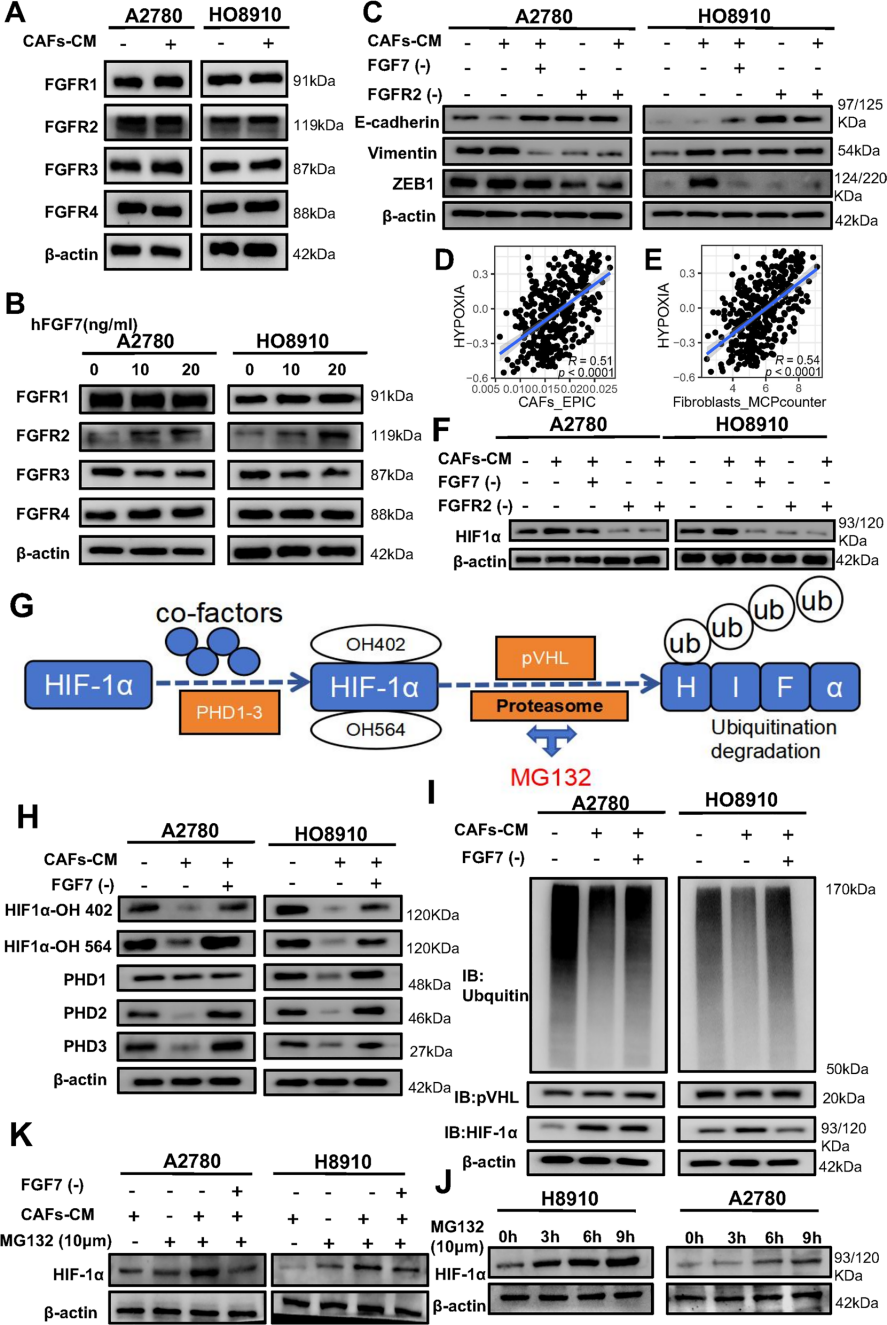
Role of FGF7-FGFR2 signaling pathway and hypoxia in ovarian cancer progression. **A** Western blotting analysis of FGFR1-4 protein expression in OC cells treated with CAFs-CM. **B** Western blotting analysis of FGFR1-4 protein expression in OC cells treated with hFGF7 at concentrations of 10 ng/ml and 20 ng/ml. **C** Western blotting analysis of EMT marker protein expression in OC cells treated with CAFs-CM, FGF7-Ab, or FGFR2 antagonists. (D-E) Scatter plot illustrating the correlation between CAFs score from two algorithms and hypoxic pathway activation status. **F** Western blotting analysis of HIF-1α protein expression in OC cells treated with CAFs-CM, FGF7-Ab, or FGFR2 antagonists. **G** Schematic representation of HIF-1α protein ubiquitination. **H** Western blotting analysis of hydroxylated-HIF-1α and PHDs expression in OC cells treated with CAFs-CM or CAFs-CM pre-incubated with FGF7-Ab. **I** Co-immunoprecipitation of ubiquitin and pVHL with HIF1α in OC cells treated with CAFs-CM or CAFs-CM by preincubation with FGF7-Ab. **J** Western blotting analysis of HIF-1α protein expression in MG132-treated OC cells at different times. **K** Western blotting analysis of HIF-1α protein expression in OC cells treated with CAFs-CM or CAFs-CM pre-incubated with FGF7-Ab and MG132

Some studies have been suggested that FGF7 holds regulatory potential over downstream signaling pathways by binding to its corresponding receptors, which include FGFR1-4. In order to corroborate the findings obtained from our single-cell data analysis, we proceeded to investigate the expression of specific FGF7 receptors in HO8910 and A2780 cells after pretreatment with conditioned media from CAFs-CM. Interestingly, our observations revealed a significant increase in the expression of FGFR2 within the cells following treatment with CAFs-CM. However, no discernible effects were observed on the expression of FGFR1, FGFR3, or FGFR4 (Fig. 6A). Further experimentation involved stimulating HO8910 and A2780 cells with varying concentrations of hFGF7, which led to the identification of a concentration-dependent relationship between hFGF7 and FGFR2 expression. Conversely, no notable impact was observed on the other FGFR receptors (Fig. 6B). Motivated by these findings, we proceeded to investigate whether inhibition of FGFR2 would influence CAF-induced EMT in OC. Encouragingly, the anticipated effects were observed as FGFR2 antagonists effectively curtailed the CAFs-CM-induced EMT in both HO8910 and A2780 cells. Moreover, FGF7 neutralizing antibodies consistently significantly inhibit CAFs-induced EMT in HO8910 and A2780 cells (Fig. 6C, Additional file 1: Fig. S5A–C).

Based on the aforementioned evidence, we propose a compelling hypothesis wherein this intricate cellular communication network (FGF7-FGFR2) undergoes remodeling within the tumor microenvironment, thereby fostering the progression of OC.

### HIF-1α is the downstream factor of FGF7 mediated progression of ovarian cancer

The hypoxic microenvironment is widely recognized as a critical factor in driving tumor progression [40]. CAFs often contribute to the development of a dense extracellular matrix, leading to intra-tumoral hypoxia [41]. In order to assess the impact of CAFs on hypoxia-related pathway, we conducted GSVA associated with hypoxia. Remarkably, we observed a robust and positive correlation between CAFs scores calculated by multiple algorithms and hypoxia scores (Fig. 6D, E), thus corroborating previous findings reported in the study. Recognizing the pivotal role of HIF-1α in orchestrating the cellular response to hypoxia [42], we sought to elucidate the molecular mechanisms underlying the regulation of OC progression by FGF7. Consequently, we investigated the influence of FGF7 on HIF-1α expression in A2780 and HO8910 cells. Analyzing clinical samples, we found a positive correlation (r = 0.239, p < 0.001) between FGF7 mRNA expression levels and HIF-1α expression (Additional file 1: Fig. S6A). Consistently, our in vitro experiments demonstrated a conspicuous upregulation of HIF-1α protein expression in A2780 and HO8910 cells upon treatment with varying concentrations of hFGF7 (Additional file 1: Fig. S6B). Moreover, we successfully verified the stimulatory effect of CAFs-CM on HIF-1α expression. Importantly, this effect was abolished when CAFs-CM was treated with FGF7-neutralizing antibodies and FGFR2 antagonist (Fig. 6F, Additional file 1: Fig. S5D). These findings strongly suggest that the influence of CAFs on HIF-1α expression is tightly associated with the presence of FGF7 in CAFs-CM. To investigate the relationship between HIF-1α and FGF7 in promoting OC progression, we conducted knockdown experiments of HIF1-α in A2780 and HO8910 cells. Notably, knockdown of HIF-1α led to a significant inhibition of OC cell proliferation (Additional file 1: Fig. S6C, D), migration (Additional file 1: Fig. S6E), and invasive ability (Additional file 1: Fig. S6F). Furthermore, we observed that CAFs-mediated EMT was also markedly suppressed upon HIF-1α knockdown (Additional file 1: Fig. S6G).

Given that HIF-1α has been reported to exhibit low expression levels, primarily due to degradation under normoxic conditions, we postulated that FGF7 might play a role in stabilizing HIF-1α in OC cells. HIF-1α undergoes degradation through hydroxylation mediated by prolyl hydroxylases (PHDs) and polyubiquitination mediated by Von-Hippel Lindau protein (pVHL). PHDs enzymatically hydroxylate HIF-1α at proline residues 402 and 564 in the presence of co-factors. Hydroxylated HIF-1α then interacts with pVHL, acting as an E3-ubiquitin ligase, leading to proteasome-dependent degradation via polyubiquitination (Fig. 6G). This process can be inhibited by MG132. We performed western blotting and assessed the expression of hydroxylated-HIF-1α at residues 402 and 564 (HIF-1α-OH 402 and HIF-1α-OH 564) as well as PHDs in HO8910 and A2780 cells under normoxic conditions. Our results demonstrated elevated expression levels of HIF-1α-OH 402, HIF-1α-OH 564, and PHDs in these cells. Subsequently, we investigated the effects of CAFs-CM on the expression of HIF-1α-OH 402, HIF-1α-OH 564, and PHDs.The induction of CAFs-CM resulted in a pronounced inhibition of their expression levels. Importantly, this effect was abolished when a FGF7-neutralizing antibody was added, implicating the involvement of FGF7 in mediating the observed effects (Fig. 6H). Importantly, Co-IP was utilized to study ubiquitin and pVHL expression. Similarly, the CAFs-induced down-regulation of HIF-1α ubiquitin and pVHL expression was reversed by the addition of FGF7-neutralizing antibody (Fig. 6I). Subsequently, we exposed A2780 and HO8910 cell lines to MG132. Western blot analysis demonstrated an increment in the HIF-1α protein concentration over this period, indicating that MG132 effectively inhibits the ubiquitin–proteasome system's ability to degrade HIF-1α in these cells (Fig. 6J). Further, OC cells were incubated with CAFs-CM, FGF7-neutralizing antibody + CAFs-CM, along with the same concentration of MG132 for 9 h. The augmentation of HIF-1α was more pronounced in the presence of CAFs-CM in conjunction with MG132, whereas the induction of HIF-1α by MG132 and CAFs-CM was little changed in the FGF7-neutralizing antibody treated cells relative to controls (Fig. 6K). These results suggest that FGF7 may promote HIF-1α expression by stabilizing the proteasome-dependent degradation pathway.

The findings observed that FGF7 upregulated the expression of HIF-1α, and knockdown of HIF-1α led to suppressed EMT facilitated by CAFs. These results provide evidence suggesting that FGF7 contributes to the stabilization of HIF-1α, thereby promoting the progression of OC through pathways involving CAFs.

### CAFs promote the direct binding of HIF-1α and ZEB1

**Fig. 7:**
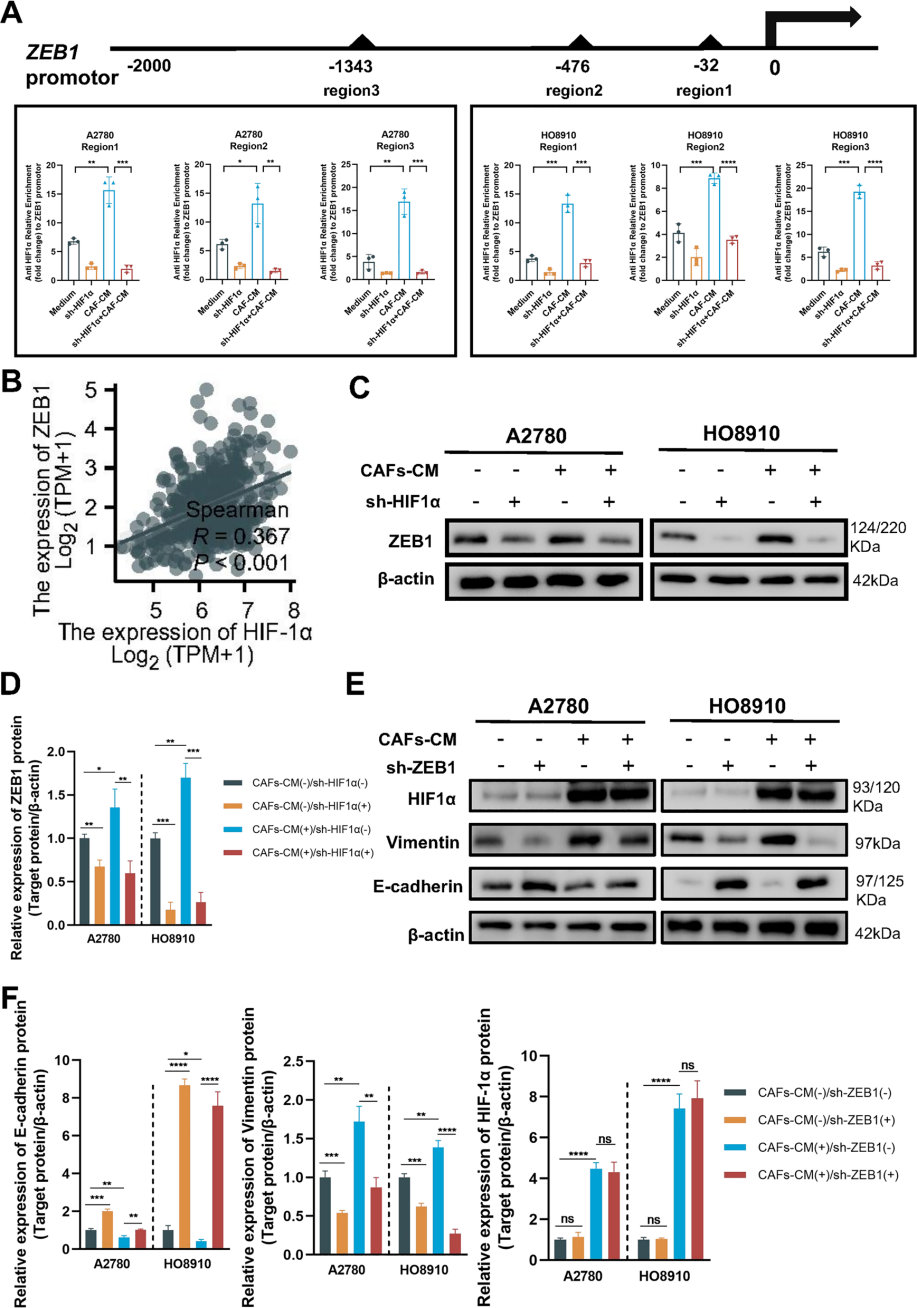
ZEB1 is a downstream factor of HIF-1α. **A** CUT&RUN assay showing the relative enrichments of DNA bound to the ZEB1 promoter at binding sites treated with CAFs-CM and corresponding HIF-1α knockdown cells. **B** Scatter plot illustrating the correlation between ZEB1 mRNA expression and HIF-1α mRNA expression. **C** Western blotting detection of ZEB1 protein expression in ovarian cancer (OC) cells with knockdown of HIF-1α. **D** Relative expression levels of ZEB1 protein by imageJ software. **E** Knockdown of ZEB1 in OC cells pretreated with or without CAFs-CM, followed by western blotting analysis of HIF-1α and EMT markers expression. **F** Relative expression levels of HIF-1α and EMT markers by imageJ software. Results are presented as the mean ± SD of three independent experiments. *p < 0.05, **p < 0.01, ***p < 0.001, ****p < 0.0001

In the context of the HIF-1α, it is well-established that ZEB1 acts as a crucial downstream factor [43–45], playing a pivotal role in EMT. To elucidate the regulatory role of HIF-1α in controlling ZEB1 expression, we employed a combination of bioinformatics analysis and CUT-RUN assay to predict and confirm three potential HIF-1α binding sites within the promoter region of ZEB1. Our results unequivocally demonstrated a significant increase in the relative enrichment of DNA bound to the ZEB1 promoter at the predicted HIF-1α binding sites in A2780 and HO8910 cells pre-treated with CAFs-CM. Importantly, this phenomenon was abrogated upon knockdown of HIF-1α (Fig. 7A). These findings establish that HIF-1α, upon evading degradation and entering the nucleus, directly regulates the transcription of ZEB1 in OC.

Furthermore, we validated this relationship between HIF-1α and ZEB1 in clinical samples, affirming a positive correlation between the expression levels of HIF-1α and ZEB1 (r = 0.367, p < 0.001), as shown in Fig. 7B. We also investigated the impact of HIF-1α knockdown on CAFs-CM-induced ZEB1 expression in A2780 and HO8910 cells. Intriguingly, our results indicated that HIF-1α significantly attenuated the induction of ZEB1 in response to CAFs-CM (Fig. 7C–E). Subsequently, we sought to explore the effects of ZEB1 inhibition on EMT by employing ZEB1 knockdown in A2780 and HO8910 cells. Western blotting (Fig. 7D) confirmed the successful knockdown of ZEB1 in these cell lines. Remarkably, our results demonstrated that ZEB1 knockdown effectively inhibited the occurrence of EMT, without exerting any influence on HIF-1α expression (Fig. 7E, F). We also found that ZEB1 knockdown (Additional file 1: Fig. S6H) resulted in a marked suppression of proliferation (Additional file 1: Fig. S6I, J), invasion (Additional file 1: Fig. S6K), and migration capacities (data not shown).

These results highlight a direct regulatory relationship between HIF-1α and ZEB1 in OC. The knockdown of HIF-1α attenuated the induction of ZEB1 by CAFs-CM, emphasizing its role in modulating ZEB1 expression. Additionally, inhibiting ZEB1 expression effectively suppressed EMT and hindered the proliferation, invasion, and migration capacities of OC cells.

### FGF7 derived from CAFs promoted tumor growth in vivo

**Fig. 8:**
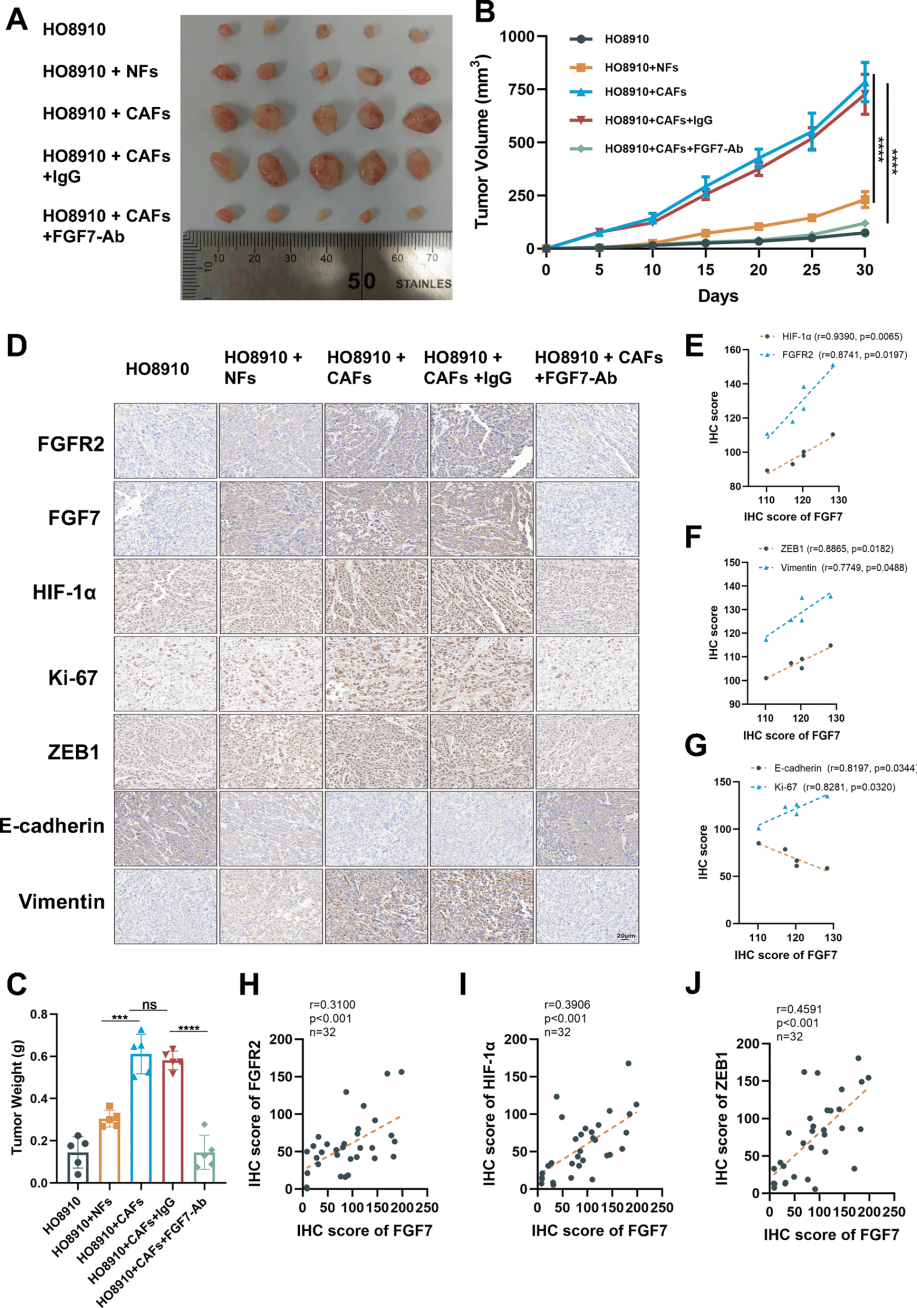
FGF7 derived from CAFs promotes tumor growth in vivo. ** A** The tumor xenografts were photographed on the 31st day of the experiment. ** B** Tumor volumes were recorded every five days. **C** Tumor weights were measured at the end of the experiment. **D** Immunohistochemistry (IHC) analysis (n = 5). **D**The correlations between FGF7 and HIF-1α, FGFR2 IHC score in xenografts specimens were evaluated with Pearson correlation analysis (n = 5). **E** The correlations between FGF7 and ZEB1, Vimentin IHC score in xenografts specimens were evaluated with Pearson correlation analysis. (n = 5). **F** The correlations between FGF7 and E-cadherin, Ki-67 IHC score in xenografts specimens were evaluated with Pearson correlation analysis (n = 5). **H**–**J** The correlations between FGF7 and E-cadherin, ZEB1, FGFR2 IHC score in OC tissues were evaluated with Pearson correlation analysis. (n = 32). *p < 0.05, **p < 0.01, ***p < 0.001, ****p < 0.0001

We established a xenograft tumor model using BALB/c nude mice. HO8910 cells were subcutaneously co-injected with CAFs/NFs in predetermined proportions. Intraperitoneal administration of FGF7-Ab commenced on the second day. The HO8910 + CAFs group exhibited significantly greater tumor weight and volume compared to the other groups. However, treatment with FGF7-Ab effectively reduced both tumor weight and volume (Fig. 8A–C). IHC analysis demonstrated that FGF7-Ab treatment attenuated the expression of FGFR2, FGF7, HIF-1α, Ki-67, ZEB1, and Vimentin, while augmenting E-cadherin expression (Fig. 8D). Additionally, IHC scores in the CAFs-CM group further indicated a prominent positive association between FGF7 expression and FGFR2, FGF7, HIF-1α, Ki-67, ZEB1, and Vimentin, along with an inverse correlation with E-cadherin expression (Fig. 8E–G). Crucially, clinical samples displayed a distinct positive correlation between FGF7 and the three pivotal molecules investigated herein: FGFR2, HIF-1α, and ZEB1 (Fig. 8H–J). In summary, these findings suggest that FGF7 derived from CAFs facilitates in vivo tumor growth and EMT.

## Discussion

The interaction between CAFs and the tumor microenvironment plays a crucial role in promoting immunosuppression, which in turn influences tumor progression and patient outcomes [41]. However, the precise mechanism underlying the regulation of OC progression by CAFs remains incompletely understood. Through comprehensive fluorescence staining analyses, we observed that regions characterized by a high density of CAFs displayed enhanced recruitment of regulatory T cells and exhibited elevated expression of α-SMA, a well-established CAF marker, in patients with advanced OC. These findings are consistent with prior studies reporting an increased density of CAFs in aggressive tumors and highlighting the association between CAFs and immune tolerance [39]. Importantly, we further established a significant correlation between CAF presence and poor prognosis through comprehensive analysis across multiple cohorts. Our findings underscore the substantial impact of CAFs in the tumor microenvironment on OC progression, based on a large sample size, multi-cohort approach, and utilization of multiple algorithms.

CAFs do not exist in isolation; instead, they interact with tumor cells, thereby promoting the malignant phenotype [2]. CAFs play a crucial role in creating a microenvironment that supports tumor growth by secreting various cytokines, growth factors, and other proteins [2]. Identifying the most critical genes derived from CAFs has become a challenging task. Currently, multi-omics investigation combined with machine learning techniques has emerged as a powerful approach for biomarker discovery across different areas of research [46, 47]. By integrating data from genomics, transcriptomics, proteomics, and metabolomics, researchers can gain a comprehensive understanding of complex biological systems and identify key molecular players that drive disease processes or serve as potential therapeutic targets [48]. To elucidate the key genes derived from CAFs, we applied advanced machine learning algorithms. Our analysis revealed that FGF7, MFAP4, DDR2, ITGBL1, COL8A1, and PDGFRA were highly expressed in CAFs. Furthermore, by utilizing a combination of 99 machine learning methods, we assigned survival risk scores to each patient. Notably, we established a final risk score with an optimal cutoff value of 77.18. Patients surpassing this threshold exhibited a poorer prognosis regardless of the cohort under consideration. Importantly, our risk score demonstrated consistent accuracy and performance across six publicly available datasets and one internal cohort, which highlights its potential for clinical application. These findings also underscore the critical significance of genes derived from CAFs in OC progression and prognosis.

In the random forest tree model, FGF7 was identified as the most important molecule. The fibroblast growth factor (FGF) family plays crucial roles in various cellular processes, including cell proliferation, differentiation, migration, and survival [49]. Among these, FGF7, also known as keratinocyte growth factor (KGF), is predominantly expressed in epithelial tissues such as the skin, lungs, gastrointestinal tract, and kidney [50]. Studies have shown that mesenchymal cells primarily produce FGF7, which then acts on adjacent epithelial cells through paracrine signaling [51]. During embryogenesis, FGF7 signaling is essential for the formation and branching morphogenesis of organs like the lungs, mammary glands, and salivary glands [52, 53]. Additionally, FGF7-mediated signaling pathways are activated in response to tissue damage or injury, triggering the regeneration process [54]. Notably, in certain epithelial cancers such as lung [55], bladder [56], and breast cancers [57], FGF7's overexpression has been observed. Moreover, Parrott et al. [50] investigated the expression and action of FGF7 in normal ovarian surface epithelium (OSE) and OC tissues. The study found high expression levels of FGF7 in normal OSE, suggesting its role as an important autocrine stimulator of OSE growth and its potential involvement in ovarian tumor growth. Consistently, our study revealed that FGF7 in the medium was more present in CAFs and less secreted in tumor cells. Fan et al. [56] conducted a study on the role of FGF7 in urothelial carcinoma of the bladder and upper urinary tract. Their results demonstrated a significant association between FGF7 overexpression and advanced disease features as well as a poor prognosis. Zhu et al. [57] investigated the role of LINC00460 in breast cancer progression. They found that LINC00460 is upregulated in breast cancer and its overexpression promotes cell viability, migration, and invasion both in vitro and in vivo. Mechanistically, Linc00460 functions as a competing endogenous RNA of FGF7 mRNA by sponging miR-489-5p. Consistent with other studies, our research also observed a significant increase in FGF7 expression in advanced OC compared to early-stage OC. Moreover, there was a significant relationship with vascular and lymphovascular invasion, and patients with high FGF7 expression had a significantly worse prognosis than those with low expression. In future studies, we will expand the clinical sample size and validate FGF7 in patients' serum to further define its diagnostic efficacy.

We observed that FGF7 derived from CAFs or hFGF7 can enhance the proliferation, invasion, and migration abilities of OC cells. FGF exerts its multiple functions by activating tyrosine kinase receptors belonging to the FGF receptor (FGFRs) family [51]. The expression of FGF and FGFRs in cancer varies depending on the tissue and context, and the diverse interactions between FGF and FGFRs significantly contribute to the complexity of FGF-FGFRs signaling during cancer progression [58]. Using a cellchat method to analyze single-cell data, we inferred that CAFs-secreted FGF7 specifically binds to FGFR2 receptors located on the surface of epithelial cells, as supported by omics-insights. This finding aligns with existing literature, which suggests that FGF7 predominantly interacts with the "b" isoform of FGFR2 (FGFR2b) and, to a lesser extent, with FGFR1 (FGFR1b) [59, 60]. From an experimental perspective, we consistently observed that CAFs-derived FGF7 or exogenous hFGF7 significantly upregulated FGFR2 expression in OC cells. The use of FGF7-neutralizing antibodies or FGFR2 inhibitors effectively counteracted the effects of CAFs-derived FGF7 or blocked FGFR2 activation, thereby mitigating the promotion of OC progression mediated by CAFs. SOX9 has been identified as a crucial regulator in cholangiocarcinoma (CCA), where it promotes plays a significant role in enhancing the expression of FGF7 and FGFR2 [58]. Of note, FGF7 acts as a key biomarker that facilitates CCA proliferation by activating FGFR2 in an autocrine pathway. In gastric cancer, increased expression of FGFR2 has been correlated with tumor depth and clinical stage [15]. The FGF7/FGFR2 signaling cascade has been found to upregulate thrombospondin 1 (THBS1), promoting invasion and migration of gastric cancer cells [15]. Notably, FGF7/FGFR2 signaling also counteracts the effects of progesterone on ER-dependent cell growth and tamoxifen response, influencing the expression and activity of estrogen receptor (ER) and progesterone receptor (PR) [61]. Additionally, high expression levels of FGFR2 and FGF7 have been associated with increased sensitivity to FGFR inhibitors in fusion-gene-positive rhabdomyosarcoma cell lines [62]. It suggests the presence of an autocrine loop involving FGF7-FGFR2. Inhibition of FGFR using NVP-BGJ398 has shown potential as a therapeutic strategy, particularly when combined with irinotecan. These findings also highlight the significance of understanding FGF7/FGFR2 signaling in various cancers and suggest avenues for further investigation in developing targeted therapeutic approaches.

HIF-1α, a transcription factor that functions as a master regulator of oxygen homeostasis, has been found to be upregulated in various cancers [43–45], including OC [63]. In normoxic conditions, HIF-1α is rapidly degraded, but under hypoxic conditions prevalent in solid tumors, its stabilization and activation occur [42]. Once activated, HIF-1α initiates a cascade of molecular events that promote angiogenesis, metabolic adaptation, and metastatic potential in cancer cells [42, 64]. In our study, a positive correlation was observed between FGF7 and HIF-1α in clinical samples. Specifically, knockdown of HIF-1α alone was found to inhibit the malignant progression of OC. Interestingly, we made the noteworthy observation that exposure to CAFs led to the activation of HIF-1α under normoxic conditions, while simultaneously inhibiting its degradation. Additionally, exogenous hFGF7 or FGF7 derived from CAFs maintained the expression of HIF-1α in the presence of normoxia within OC cells. Remarkably, knocking down HIF-1α in OC cells significantly suppressed CAFs induced EMT and impeded tumor-promoting effects. These findings substantiate the crucial involvement of the HIF-1α signaling pathway in modulating the phenotypic effects induced by CAFs-derived FGF7.

During EMT, cells undergo a phenotypic switch from an epithelial to a mesenchymal state, characterized by the loss of epithelial markers and acquisition of mesenchymal markers, along with enhanced migratory and invasive capabilities [65]. ZEB1, as a key transcription factor, has emerged as a critical regulator of EMT [66, 67]. Our study demonstrates the regulatory role of HIF-1α in transcription and expression through direct binding to ZEB1. Importantly, knockdown of ZEB1 in OC cells exerts a pronounced inhibitory effect on CAFs-induced EMT.

The presented study has several limitations that should be considered. Firstly, the generalizability of the findings may be limited due to the predominant focus on bulk transcriptomic datasets and in vitro experiments. Although these approaches provide valuable insights, they may not fully capture the complexity and heterogeneity of OC in clinical settings. Therefore, caution should be exercised when extrapolating the results to encompass all subtypes. In terms of molecular pathways, while the study identifies the FGF7/HIF-1α pathway as a mechanism underlying OC progression, it does not extensively explore other potential molecular pathways involved, such as the lack of investigation into the phosphorylation levels of FGFR2 substrates. Moreover, although the study demonstrates the efficacy of neutralizing antibodies targeting CAFs-derived FGF7 in reducing tumor growth in vivo, more research is needed to evaluate the long-term safety, potential side effects, and optimal dosing strategies of such therapeutic interventions. Additionally, assessing the efficacy of these interventions in combination with existing treatment modalities is crucial for evaluating their clinical applicability.

## Conclusion

Our study employs a multi-omics approach to elucidate the pivotal molecular factor FGF7, which is derived from CAFs. To stratify patients based on their survival risk, we propose a key cut-off value of 77.18, identified through machine learning analysis of FGF7 expression along with other genes. Crucially, we introduce a novel mechanism wherein FGF7 serves as a critical regulatory factor facilitating crosstalk between CAFs and cancer cells, thereby promoting the progression of OC via activation of the HIF1α/EMT signaling axis.

### Supplementary Information

**Additional file 1: Figure S1.** Weighted Gene Co-expression Network Analysis and correlation analysis of modules and CAFs scores. **Figure S2.** Kaplan-Meier survival analysis plot of different cohorts. **Figure S3.** Effects of FGF7 knockdown and different conditioned medium on biological function of ovarian cancer. **Figure S4.** Exploration of CAFs subtypes in ovarian cancer. **Figure S5.** Statistics of gray values of target proteins in different subgroups in WB experiments. **Figure S6.** Effects of HIF-1α knockdown on biological function of ovarian cancer. **Table S1.** A list of primers used in this study. **Table S2.** Basic information about the bulk transcriptome cohorts. **Table S3.** The performance of 99 predictive models in training and testing cohorts.

## Data Availability

The following information was supplied regarding data availability: Data is available at the TCGA (https://portal.gdc.cancer.gov/), GEO database (https://www.ncbi.nlm.nih.gov/geo/), and CCLE database (https://sites.broadinstitute.org/ccle/). The detailed data that support the findings of this study are available from the corresponding author upon reasonable request.
