# Supplementary material for: Cancer-associated fibroblast-secreted FGF7 as an ovarian cancer progression promoter
Source: J Transl Med. 2024 Mar 15;22:280. doi: 10.1186/s12967-024-05085-y (PMC10941588; doi:10.1186/s12967-024-05085-y)
Supplement: Supplementary file 1 — Additional file 1: Figure S1. Weighted Gene Co-expression Network Analysis and correlation analysis of modules and CAFs scores. Figure S2. Kaplan-Meier survival analysis plot of different cohorts. Figure S3. Effects of FGF7 knockdown and different conditioned medium on biological function of ovarian cancer. Figure S4. Exploration of CAFs subtypes in ovarian cancer. Figure S5. Statistics of gray values of target proteins in different subgroups in WB experiments. Figure S6. Effects of HIF-1α knockdown on biological function of ovarian cancer. Table S1. A list of primers used in this study. Table S2. Basic information about the bulk transcriptome cohorts. Table S3. The performance of 99 predictive models in training and testing cohorts. [file 12967_2024_5085_MOESM1_ESM.pdf]

Figure S1. Weighted Gene Co-expression Network Analysis and correlation analysis of modules and CAFs scores.

(A) Determination of soft threshold power in the RNA-seq (upper panel) and GPL570 (lower panel) cohorts.

(B)Clustering analysis of modules in the RNA-seq (upper panel) and GPL570 (lower panel) cohorts.

(C) Heat map displaying the correlation between modules and traits in the RNA-seq (left panel) and GPL570 (right panel) cohorts.

(D) Scatter plot illustrating the correlation between Module Membership (MM) and Gene Significance (GS) of the blue module. The sequences from top to bottom represent CAF scores calculated by MCPcounter in the RNA-seq cohort, CAF scores calculated by EPIC in the RNA-seq cohort, CAF scores calculated by MCPcounter in the GPL570 cohort, and CAF scores calculated by EPIC in the GPL570 cohor.

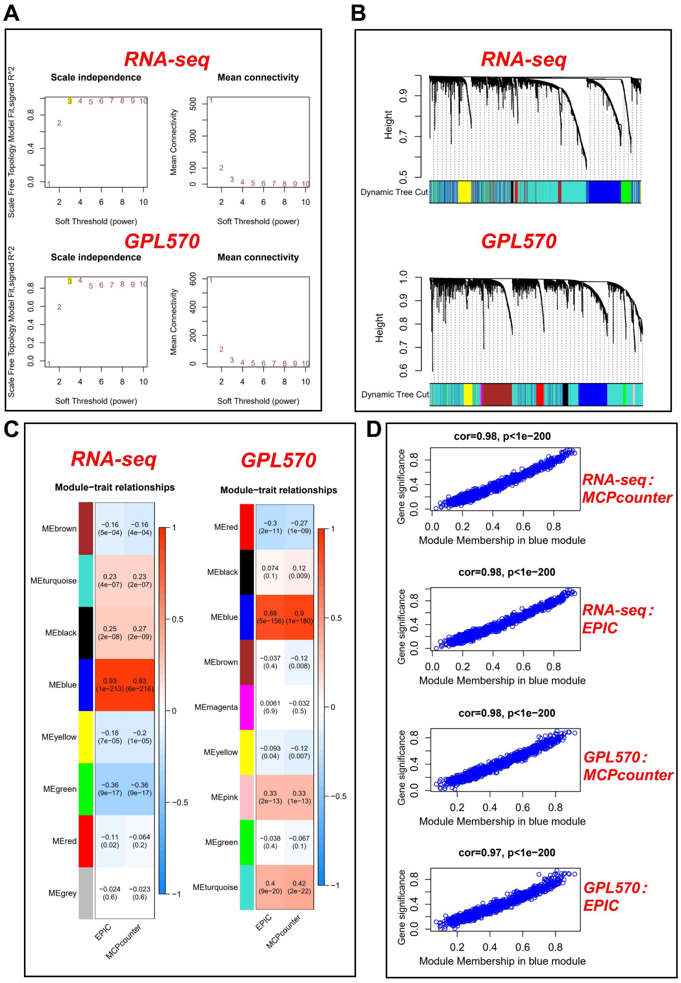

Figure S2. Kaplan-Meier survival analysis plot of different cohorts.

(A) Kaplan-Meier survival analysis of the GPL7759 cohort.

(B) Kaplan-Meier survival analysis of the GPL96 cohort.

(C) Kaplan-Meier survival analysis of the GPL2986 cohort.

(D) Kaplan-Meier survival analysis of the GPL6480 cohort.

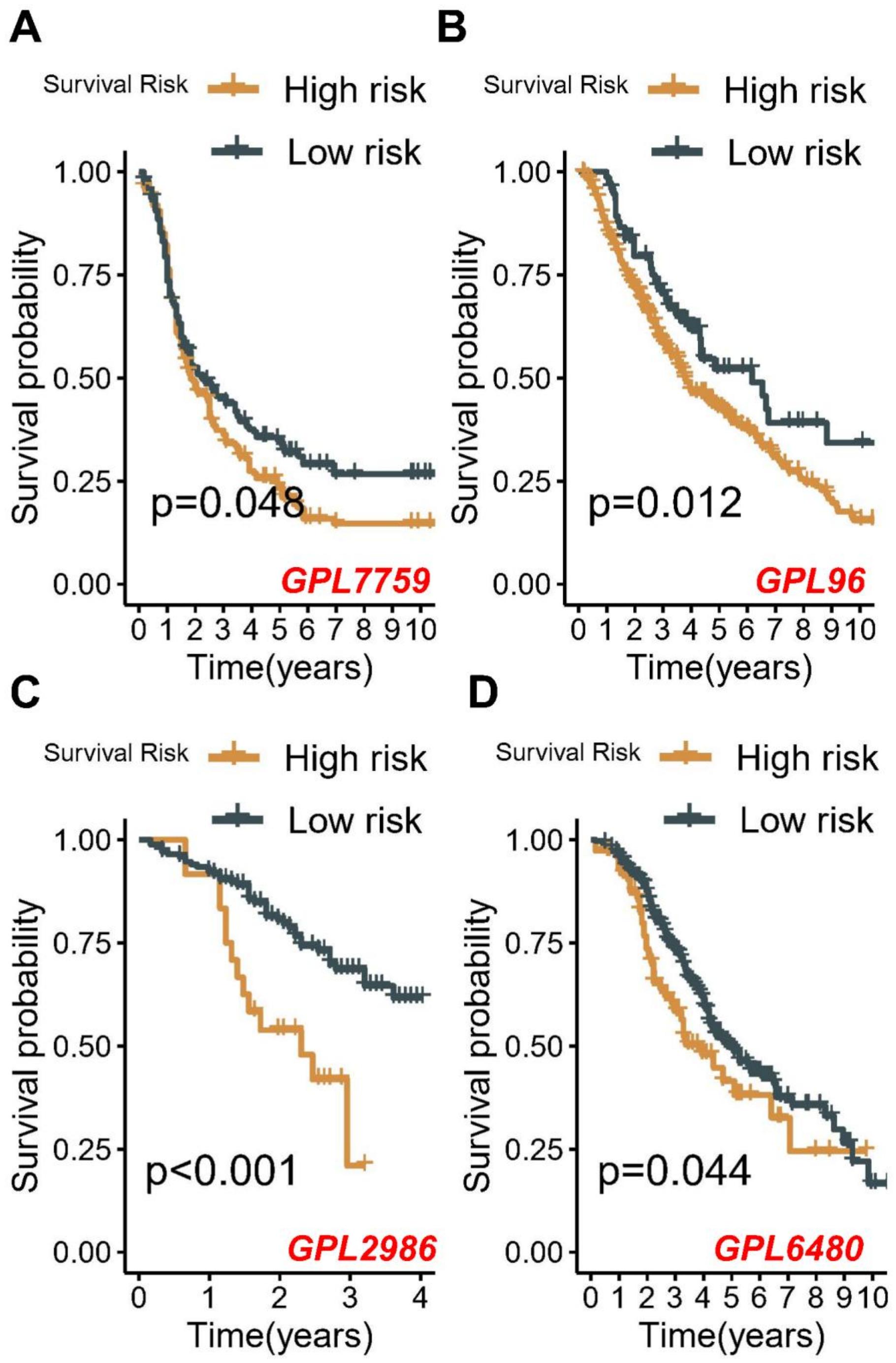

Figure S3. Effects of FGF7 knockdown and different conditioned medium on biological function of ovarian cancer.

(A)Wound healing assay used to measure OC cells migration ability after treatment with different conditioned medium (100× magnification).

(B) Cell invasion ability evaluated by Transwell assay after 48 hours in OC cells after treatment with different conditioned medium (200× magnification).

(C) Cell viability of HO8910 cells measured by CCK8 assay after treatment with different conditioned medium.

(D) Cell viability of A2780 cells measured by CCK8 assay after treatment with different conditioned medium.

(E) The expression of FGF7 in HO8910 cells transfected with different si-RNA was detected by RT-qPCR.

(F) The expression of FGF7 in A2780 cells transfected with different si-RNA was detected by RT-qPCR.

(G) CCK8 assay measuring the cell viability of A2780 cells after FGF7 knockdown.

(H) CCK8 assay measuring the cell viability of HO8910 cells after FGF7 knockdown..

(I) Wound healing assay used to measure OC cells migration ability after FGF7 knockdown (100× magnification).

(J) Cell invasion ability evaluated by Transwell assay after 48 hours in OC cells after FGF7 knockdown (200× magnification).

Results are presented as the mean ± SD of three independent experiments. $*P < 0.05$, $**P < 0.01$, $***P < 0.001$, $****P < 0.0001$, ns not significant.

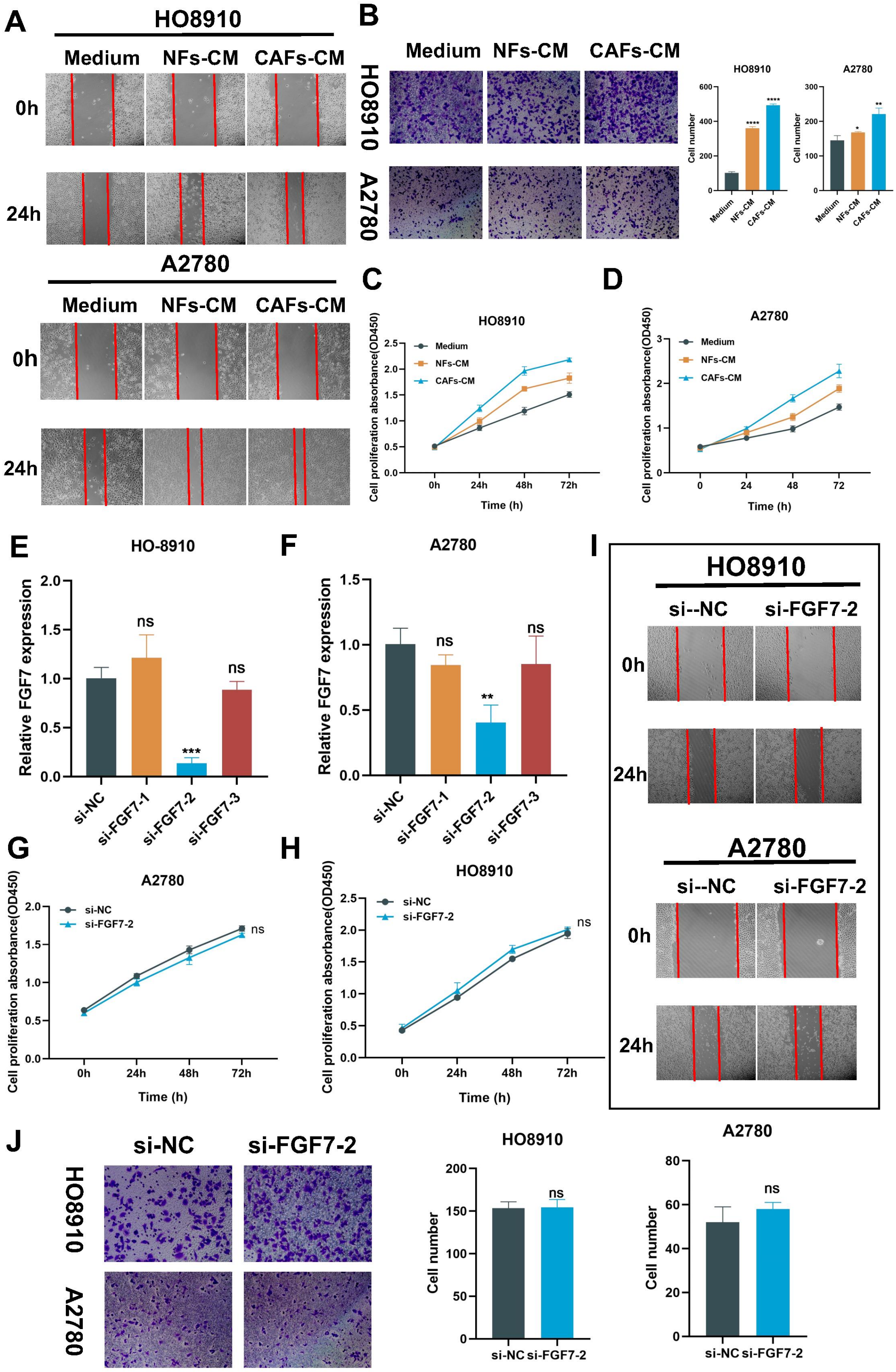

A
HO8910
Medium
NFs-CM
CAFs-CM
0h
24h
A2780
Medium
NFs-CM
CAFs-CM
0h
24h
B
Medium
NFs-CM
CAFs-CM
HO8910
A2780
HO8910
A2780
Cell number
C
HO8910
Medium
NFs-CM
CAFs-CM
Cell proliferation absorbance(OD450)
Time (h)
D
A2780
Medium
NFs-CM
CAFs-CM
Cell proliferation absorbance(OD450)
Time (h)
E
HO-8910
Relative FGF7 expression
ns
***
ns
si-NC
si-FGF7-1
si-FGF7-2
si-FGF7-3
F
A2780
Relative FGF7 expression
ns
**
ns
si-NC
si-FGF7-1
si-FGF7-2
si-FGF7-3
I
HO8910
si--NC
si-FGF7-2
0h
24h
A2780
si--NC
si-FGF7-2
0h
24h
G
A2780
si-NC
si-FGF7-2
ns
Cell proliferation absorbance(OD450)
Time (h)
H
HO8910
si-NC
si-FGF7-2
ns
Cell proliferation absorbance(OD450)
Time (h)
J
si-NC
si-FGF7-2
HO8910
A2780
HO8910
Cell number
ns
si-NC
si-FGF7-2
A2780
Cell number
ns
si-NC
si-FGF7-2

Figure S4. Exploration of CAFs subtypes in ovarian cancer.

(A) Uniform Manifold Approximation and Projection (UMAP) plot illustrating major cell populations, with dots colored to represent different cell types in all CAFs.

(B) Histogram of the distribution of different CAFs subtypes in the samples.

(C) Heat map showing similarities between the four CAF subtypes of OC and the CAF subtypes observed in pan-cancer.

(D) Bubble plots of the top five specificity markers for different CAFs subtypes.

(E) Ridge map showing the expression of alpha-smooth muscle actin (ACTA2) in different CAF subtypes.

(F) Ridge map showing the expression of fibroblast growth factor 7 (FGF7) in different CAF subtypes.

(G) Ridge map showing the expression of fibroblast growth factor receptor 2 (FGFR2) in different CAF subtypes.

(H) Violin plots of the distribution of epithelial cell EMT scores in different samples.

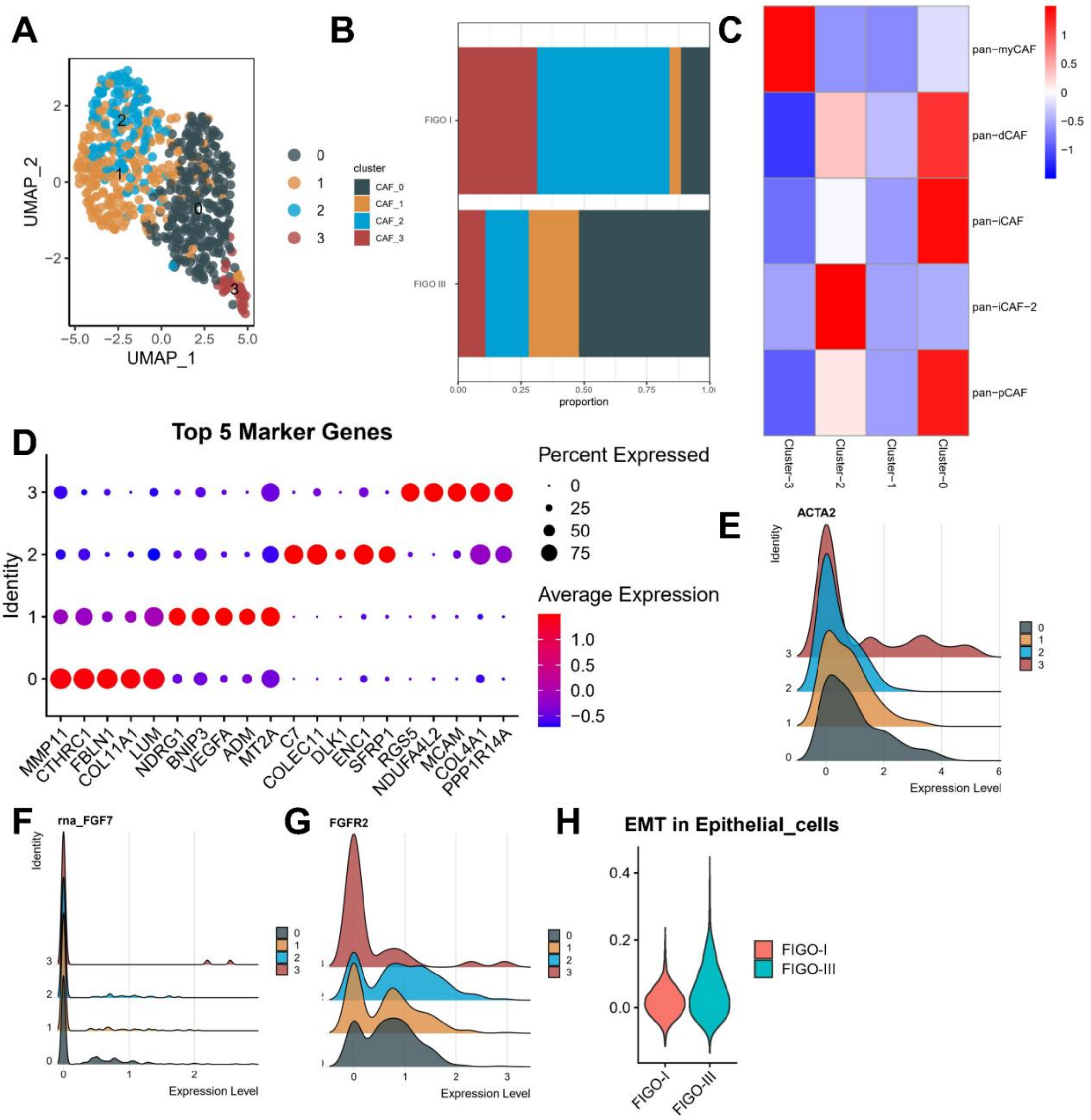

A
UMAP_1
UMAP_2
B
FIGO I
FIGO III
proportion
cluster
CAF_0
CAF_1
CAF_2
CAF_3
C
pan−myCAF
pan−dCAF
pan−iCAF
pan−iCAF−2
pan−pCAF
Cluster−3
Cluster−2
Cluster−1
Cluster−0
D
Top 5 Marker Genes
Identity
Percent Expressed
Average Expression
MMP11
CTHRC1
FBLN1
COL11A1
LUM
NDRG1
BNIP3
VEGFA
ADM
MT2A
C7
COLEC11
DLK1
ENC1
SFRP1
RGS5
NDUFA4L2
MCAM
COL4A1
PPP1R14A
E
ACTA2
Expression Level
F
rna_FGF7
G
FGFR2
H
EMT in Epithelial_cells
FIGO-I
FIGO-III

Figure S5. Statistics of gray values of target proteins in different subgroups in WB experiments.

(A) Relative expression levels of E-cadherin protein.

(B) Relative expression levels of ZEB1 protein.

(C) Relative expression levels of Vimentin protein.

(D) Relative expression levels of HIF-1α protein.

Results are presented as the mean ± SD of three independent experiments. *P < 0.05, **P < 0.01, ***P < 0.001, ****P < 0.0001, ns not significant.

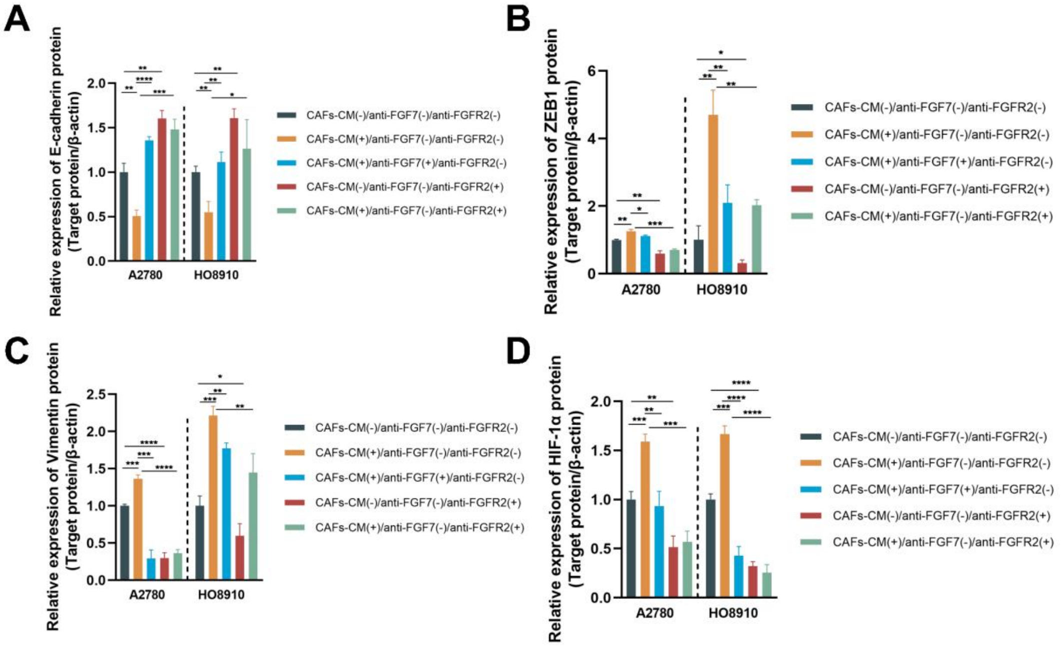

Figure S6. Effects of HIF-1α knockdown on biological function of ovarian cancer.

(A) Scatter plot illustrating the correlation between FGF7 mRNA expression and HIF-1α mRNA expression.

(B) Western blotting analysis of HIF-1α protein expression in OC cells treated with hFGF7 at concentrations of 10 ng/ml and 20 ng/ml.

(C) CCK8 assay measuring the cell viability of HO8910 cells after HIF-1α knockdown.

(D) CCK8 assay measuring the cell viability of A2780 cells after HIF-1α knockdown.

(E) Transwell assay validating the cell invasion ability of HO8910 and A2780 cells after 48 hours of HIF-1α knockdown (200× magnification).

(F) Wound healing assay used to measure cell migration ability after HIF-1α knockdown for 24 hours (100× magnification).

(G) Western blotting detection of EMT markers protein expression in OC cells with knockdown of HIF-1α.

(H) The expression of ZEB1 in HO8910 and A2780 cells transfected with sh-ZEB1 was detected by Western blotting.

(I) CCK8 assay measuring the cell viability of HO8910 cells after ZEB1 knockdown.

(J) CCK8 assay measuring the cell viability of A2780 cells after ZEB1 knockdown.

(K) Transwell assay validating the cell invasion ability of HO8910 and A2780 cells after 48 hours of ZEB1 knockdown (200× magnification).

Results are presented as the mean ± SD of three independent experiments. *$P < 0.05$, **$P < 0.01$, ***$P < 0.001$, ****$P < 0.0001$, ns not significant.

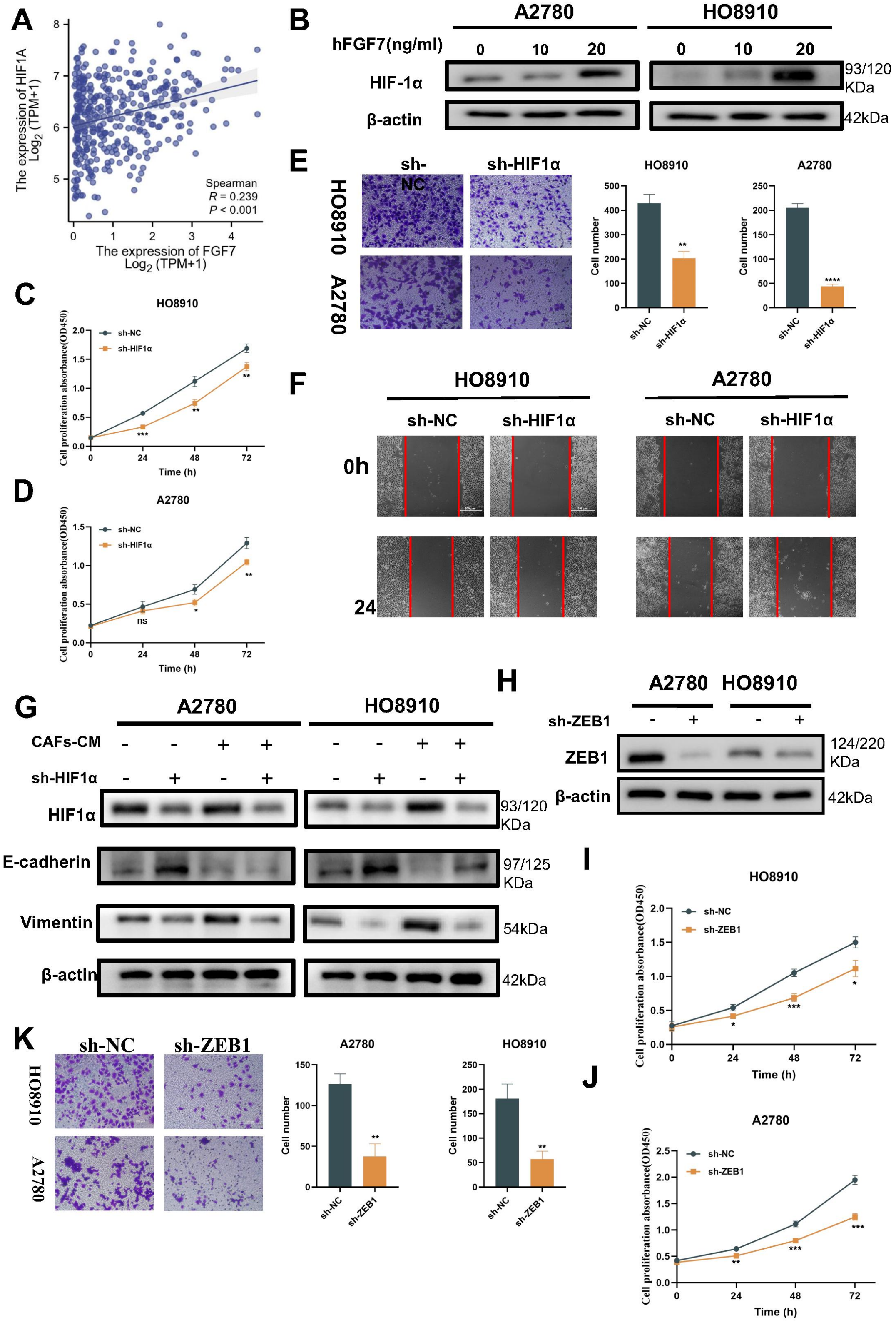

A
The expression of HIF1A Log2 (TPM+1)
The expression of FGF7 Log2 (TPM+1)
Spearman
R = 0.239
P < 0.001
B
A2780
HO8910
hFGF7(ng/ml)
0 10 20
0 10 20
HIF-1α
β-actin
93/120 KDa
42kDa
C
HO8910
sh-NC
sh-HIF1α
Cell proliferation absorbance(OD450)
Time (h)
D
A2780
sh-NC
sh-HIF1α
Cell proliferation absorbance(OD450)
Time (h)
ns
E
sh-NC
sh-HIF1α
HO8910
A2780
Cell number
F
HO8910
A2780
sh-NC
sh-HIF1α
0h
24
G
A2780
HO8910
CAFs-CM
sh-HIF1α
HIF1α
E-cadherin
Vimentin
β-actin
93/120 KDa
97/125 KDa
54kDa
42kDa
H
A2780
HO8910
sh-ZEB1
ZEB1
β-actin
124/220 KDa
42kDa
I
HO8910
sh-NC
sh-ZEB1
Cell proliferation absorbance(OD450)
Time (h)
J
A2780
sh-NC
sh-ZEB1
Cell proliferation absorbance(OD450)
Time (h)
K
sh-NC
sh-ZEB1
HO8910
A2780
Cell number

Table S1. A list of primers used in this study.

Gene	Forward sequence (5' to 3')	Reverse sequence (5' to 3')
ZEB1-32	GGACTGGAAAGCGGAAACTT	CGAGGTGTCTGGGAGTTGG
ZEB1-476	CGGGGGAGGGGGACTGGAAAGC	AGGCCTCCTGGAAACGGTGCCG
ZEB1-1343	GCTGGGCCAGGCTGCTTTGCA	GGACACCTGACCTTCCGACG
β-actin	CTACCTCATGAAGATCCTCACC	AGTTGAAGGTAGTTTCGTGGAT
α-SMA	CTATGAGGGCTATGCCTTGCC	GCTCAGCAGTAGTAACGAAGGA
Vimentin	GACGCCATCAACACCGAGTT	CTTTGTCGTTGGTTAGCTGGT
FGF7	AGTTGCACCAGGCAGACAAC	TAAGTTCAGTTGCTGTGACGC

Table S2. Basic information about the bluk transcriptome cohorts.

Cohort	Datasets	Platform	Samples	PMID
Meta RNA-seq Cohort	TCGA_OV	Illumina HiSeq 2000	364	21720365
	ICGC_OV	Illumina HiSeq 2000	111	30877282
Meta GPL96 Cohort	GSE14764	Affymetrix Human Genome U133A Array	68	19294737
	GSE26712	Affymetrix Human Genome U133A Array	184	18593951
	GSE23554	Affymetrix Human Genome U133A Array	28	21849418
	GSE3149	Affymetrix Human Genome U133A Array	115	16273092
Meta GPL570 Cohort	GSE19829	Affymetrix Human Genome U133 Plus 2.0 Array	28	20547991
	GSE30161	Affymetrix Human Genome U133 Plus 2.0 Array	47	22348014
	GSE63885	Affymetrix Human Genome U133 Plus 2.0 Array	70	24478986
	GSE26193	Affymetrix Human Genome U133 Plus 2.0 Array	77	30244973
	GSE9891	Affymetrix Human Genome U133 Plus 2.0 Array	197	18698038
	GSE18520	Affymetrix Human Genome U133 Plus 2.0 Array	53	19962670
GPL2986 Cohort	GSE49997	ABI Human Genome Survey Microarray Version 2	193	22497737
GPL14951 Cohort	GSE140082	Illumina HumanHT-12 WG-DASL V4.0 R2 expression beadc hip	273	28159814
Meta GPL6480 Cohort	GSE17260	Agilent-014850 Whole Human Genome Microarray 4x44K	106	20300634
	GSE32063	Agilent-014850 Whole Human Genome Microarray 4x44K	40	22241791
	GSE32062	Agilent-014850 Whole Human Genome Microarray 4x44K	259	22241791
GPL7759 Cohort	GSE13876	Operon human v3 ~35K 70-mer two-color oligonucleotide mic roarrays	413	19192944

Table S3: The performance of 99 predictive models in training and testing cohorts.

Model	RNA-seq	GPL96	GPL570	GPL2986	GPL6480	GPL7759
Lasso+StepCox[both]	0.5703	0.5671	0.6377	0.5964	0.5213	0.5250
survivalSVM	0.5801	0.6089	0.6484	0.6516	0.5358	0.5449
CoxBoost+survivalSVM	0.5849	0.6048	0.6516	0.6288	0.5290	0.5554
Ridge	0.5788	0.6000	0.6448	0.6408	0.5349	0.5408
Lasso+survivalSVM	0.5836	0.6051	0.6539	0.6188	0.5257	0.5482
SuperPC	0.5762	0.6075	0.6404	0.6651	0.5390	0.5404
CoxBoost+Ridge	0.5794	0.5877	0.6459	0.6378	0.5295	0.5542
Enet[alpha=0.1]	0.5783	0.5858	0.6451	0.6244	0.5285	0.5412
CoxBoost+Enet[alpha=0.1]	0.5788	0.5813	0.6462	0.6255	0.5283	0.5462
Enet[alpha=0.2]	0.5769	0.5822	0.6454	0.6200	0.5275	0.5397
Enet[alpha=0.3]	0.5764	0.5806	0.6461	0.6157	0.5275	0.5387
CoxBoost+Enet[alpha=0.3]	0.5771	0.5787	0.6461	0.6190	0.5288	0.5406
CoxBoost+Enet[alpha=0.2]	0.5778	0.5798	0.6458	0.6222	0.5288	0.5429
Enet[alpha=0.4]	0.5765	0.5802	0.6458	0.6152	0.5278	0.5386
CoxBoost+Enet[alpha=0.4]	0.5767	0.5781	0.6459	0.6187	0.5293	0.5404
Lasso+CoxBoost	0.5761	0.5804	0.6459	0.6158	0.5284	0.5385
Enet[alpha=0.5]	0.5763	0.5797	0.6457	0.6157	0.5282	0.5382
CoxBoost	0.5752	0.5741	0.6428	0.6146	0.5289	0.5362
CoxBoost+Enet[alpha=0.5]	0.5769	0.5784	0.6456	0.6188	0.5290	0.5398
Enet[alpha=0.6]	0.5762	0.5794	0.6458	0.6161	0.5284	0.5382
CoxBoost+Enet[alpha=0.6]	0.5768	0.5780	0.6454	0.6179	0.5293	0.5394
CoxBoost+Enet[alpha=0.7]	0.5768	0.5778	0.6455	0.6180	0.5294	0.5393
CoxBoost+Enet[alpha=0.8]	0.5766	0.5780	0.6458	0.6177	0.5295	0.5399
Enet[alpha=0.8]	0.5761	0.5786	0.6452	0.6152	0.5286	0.5379
Enet[alpha=0.9]	0.5763	0.5793	0.6460	0.6155	0.5286	0.5384
Lasso	0.5760	0.5791	0.6457	0.6157	0.5290	0.5382
Enet[alpha=0.7]	0.5762	0.5793	0.6461	0.6157	0.5283	0.5382
CoxBoost+Enet[alpha=0.9]	0.5766	0.5780	0.6459	0.6176	0.5294	0.5401
CoxBoost+Lasso	0.5765	0.5781	0.6458	0.6180	0.5295	0.5399
Lasso+plsRcox	0.5800	0.5947	0.6476	0.6193	0.5266	0.5421
CoxBoost+plsRcox	0.5797	0.5906	0.6452	0.6443	0.5301	0.5561
CoxBoost+StepCox[forward]	0.5763	0.5788	0.6470	0.6162	0.5286	0.5390
Lasso+StepCox[forward]	0.5757	0.5803	0.6459	0.6154	0.5282	0.5384
RSF+survivalSVM	0.5801	0.6089	0.6484	0.6516	0.5358	0.5449
CoxBoost+SuperPC	0.5793	0.5971	0.6464	0.6551	0.5334	0.5576
StepCox[forward]	0.5780	0.5688	0.6359	0.6039	0.5184	0.5392
plsRcox	0.5768	0.6031	0.6422	0.6552	0.5373	0.5396
RSF+Ridge	0.5788	0.6000	0.6448	0.6410	0.5349	0.5408

RSF+Enet[alpha=0.1]	0.5783	0.5860	0.6451	0.6244	0.5286	0.5413
Lasso+SuperPC	0.5788	0.6015	0.6493	0.6224	0.5290	0.5410
RSF+plsRcox	0.5768	0.6031	0.6422	0.6552	0.5373	0.5396
RSF+StepCox[forward]	0.5780	0.5688	0.6359	0.6039	0.5184	0.5392
RSF+Enet[alpha=0.2]	0.5767	0.5822	0.6453	0.6197	0.5274	0.5395
RSF+Enet[alpha=0.3]	0.5764	0.5805	0.6459	0.6168	0.5276	0.5388
RSF+Enet[alpha=0.6]	0.5762	0.5790	0.6457	0.6165	0.5286	0.5377
RSF+Lasso	0.5755	0.5747	0.6439	0.6110	0.5284	0.5355
RSF+Enet[alpha=0.7]	0.5764	0.5790	0.6453	0.6161	0.5288	0.5380
RSF+Enet[alpha=0.5]	0.5763	0.5797	0.6459	0.6159	0.5281	0.5384
RSF+CoxBoost	0.5762	0.5790	0.6447	0.6175	0.5293	0.5386
RSF+Enet[alpha=0.9]	0.5762	0.5792	0.6457	0.6152	0.5288	0.5382
RSF+Enet[alpha=0.4]	0.5764	0.5796	0.6459	0.6154	0.5280	0.5382
RSF+Enet[alpha=0.8]	0.5763	0.5793	0.6460	0.6155	0.5286	0.5383
RSF+StepCox[both]	0.5703	0.5671	0.6377	0.5964	0.5213	0.5250
RSF+StepCox[backward]	0.5703	0.5671	0.6377	0.5964	0.5213	0.5250
StepCox[both]+Ridge	0.5703	0.5690	0.6381	0.5988	0.5202	0.5259
StepCox[backward]+Ridge	0.5701	0.5688	0.6381	0.5985	0.5200	0.5260
StepCox[both]+plsRcox	0.5691	0.5697	0.6386	0.5962	0.5187	0.5281
StepCox[backward]+plsRcox	0.5691	0.5697	0.6386	0.5962	0.5187	0.5281
StepCox[both]+Enet[alpha=0.9]	0.5702	0.5669	0.6377	0.5962	0.5212	0.5247
StepCox[backward]+Enet[alpha=0.9]	0.5702	0.5669	0.6377	0.5962	0.5212	0.5247
StepCox[both]+Enet[alpha=0.1]	0.5705	0.5678	0.6382	0.5963	0.5208	0.5255
StepCox[backward]+Enet[alpha=0.1]	0.5703	0.5676	0.6381	0.5959	0.5207	0.5256
StepCox[both]+Enet[alpha=0.8]	0.5702	0.5669	0.6377	0.5962	0.5213	0.5246
StepCox[backward]+Enet[alpha=0.8]	0.5702	0.5669	0.6377	0.5962	0.5213	0.5246
StepCox[both]+Enet[alpha=0.2]	0.5703	0.5672	0.6379	0.5962	0.5212	0.5253
StepCox[backward]+Enet[alpha=0.2]	0.5703	0.5672	0.6379	0.5960	0.5213	0.5253
StepCox[both]+Lasso	0.5702	0.5669	0.6377	0.5963	0.5212	0.5247
StepCox[backward]+Lasso	0.5702	0.5669	0.6377	0.5963	0.5212	0.5247
StepCox[both]+Enet[alpha=0.6]	0.5702	0.5669	0.6377	0.5962	0.5212	0.5247
StepCox[backward]+Enet[alpha=0.6]	0.5702	0.5669	0.6377	0.5962	0.5212	0.5247
CoxBoost+GBM	0.6565	0.6076	0.6534	0.6244	0.4874	0.4994
StepCox[both]+Enet[alpha=0.7]	0.5702	0.5669	0.6377	0.5963	0.5213	0.5247
StepCox[backward]+Enet[alpha=0.7]	0.5702	0.5669	0.6377	0.5963	0.5213	0.5247
Lasso+StepCox[backward]	0.5703	0.5671	0.6377	0.5964	0.5213	0.5250
StepCox[both]	0.5703	0.5671	0.6377	0.5964	0.5213	0.5250
StepCox[backward]	0.5703	0.5671	0.6377	0.5964	0.5213	0.5250
CoxBoost+StepCox[both]	0.5703	0.5671	0.6377	0.5964	0.5213	0.5250
CoxBoost+StepCox[backward]	0.5703	0.5671	0.6377	0.5964	0.5213	0.5250
StepCox[both]+Enet[alpha=0.4]	0.5702	0.5670	0.6377	0.5963	0.5214	0.5249
StepCox[backward]+Enet[alpha=0.4]	0.5702	0.5670	0.6377	0.5963	0.5214	0.5249
StepCox[both]+Enet[alpha=0.3]	0.5703	0.5671	0.6377	0.5964	0.5214	0.5250

StepCox[backward]+Enet[alpha=0.3]	0.5702	0.5670	0.6377	0.5964	0.5214	0.5249
StepCox[both]+CoxBoost	0.5703	0.5667	0.6374	0.5960	0.5214	0.5245
StepCox[backward]+CoxBoost	0.5703	0.5670	0.6376	0.5967	0.5211	0.5247
StepCox[both]+Enet[alpha=0.5]	0.5702	0.5669	0.6378	0.5962	0.5214	0.5248
StepCox[backward]+Enet[alpha=0.5]	0.5702	0.5669	0.6377	0.5962	0.5212	0.5246
CoxBoost+RSF	0.8572	0.6071	0.6495	0.6018	0.5110	0.5029
RSF+SuperPC	0.5762	0.6075	0.6404	0.6651	0.5390	0.5404
RSF	0.8781	0.6043	0.6416	0.5738	0.4775	0.5043
Lasso+GBM	0.6719	0.6048	0.6579	0.6142	0.5151	0.5021
RSF+GBM	0.6774	0.6056	0.6582	0.5562	0.5263	0.4990
GBM	0.6738	0.6033	0.6531	0.5604	0.5231	0.4968
StepCox[both]+survivalSVM	0.5685	0.5719	0.6390	0.5955	0.5182	0.5296
StepCox[backward]+survivalSVM	0.5685	0.5719	0.6390	0.5955	0.5182	0.5296
Lasso+RSF	0.8593	0.5984	0.6467	0.5838	0.5076	0.4969
StepCox[both]+GBM	0.6351	0.5400	0.5700	0.5724	0.4922	0.5000
StepCox[backward]+GBM	0.6318	0.5400	0.5700	0.5696	0.4919	0.5000
StepCox[both]+RSF	0.8174	0.5456	0.5638	0.5263	0.5055	0.5000
StepCox[backward]+RSF	0.8193	0.5456	0.5638	0.5258	0.5054	0.5000
